# Supplementary material for: A distributed differential game approach to trajectory planning for offshore wind farm inspection
Source: PLoS One. 2026 Mar 26;21(3):e0344989. doi: 10.1371/journal.pone.0344989 (PMC13021010; doi:10.1371/journal.pone.0344989)
Supplement: S1 Appendix — (PDF) [file pone.0344989.s001.pdf]

S1 Appendix

For the sake of clarity in the proof of the Proposition 1, we introduce the following detailed derivation of the adjoint system (33).

Consider a global cost function that consists of a weighted sum of the individual cost functions of each UAV:

$$J = \sum_{i=1}^{N} J_i. \tag{1}$$

Define the global Hamiltonian function:

$$H \triangleq \sum_{i=1}^{N} \left(\|Y_i(z_i)\|_{Q_i}^2 + \|u_i(t)\|_{R_i}^2 + \sum_{j \in \mathcal{N}_i} \|u_{ij}\|_{R_{ij}}^2 \right) + \lambda^T \left(Az + \sum_{i=1}^{N} \left(B_i u_i + \hat{\tilde{g}}_i \right) \right). \tag{2}$$

where $\lambda \in \mathbb{R}^{8N}$.

According to the PMP, the optimal control u^* satisfies:

$$\frac{\partial H}{\partial u_i} = 0. \tag{3}$$

Then,

$$u_i^* = -R_i^{-1} B_i^T \lambda. \tag{4}$$

The state equation and costate equation are respectively:

$$\dot{z} = \frac{\partial H}{\partial \lambda} = Az + \sum_{i=1}^{N} \left(B_i u_i + \hat{\tilde{g}}_i \right). \tag{5}$$

$$\dot{\lambda} = -\frac{\partial H}{\partial z} = -\sum_{i=1}^{N} \frac{\partial}{\partial z} \left(\|Y_i(z_i)\|_{Q_i}^2 \right) - A^T \lambda. \tag{6}$$

We perform a quadratic approximation around the Nash equilibrium trajectory z^*, let $\hat{\tilde{z}} = z - z^*$, then $Y_i(z_i)$ can be approximated as:

$$Y_i(z_i) \approx Y_i(z_i^*) + \nabla Y_i(z_i^*)^T \hat{\tilde{z}}_i + \frac{1}{2} \hat{\tilde{z}}_i^T \nabla Y_i^2(z_i^*)^T \hat{\tilde{z}}_i. \tag{7}$$

Therefore,

$$\|Y_i(z_i)\|_{Q_i}^2 = \Xi + \hat{\tilde{z}}_i^T \nabla Y_i(z_i^*) Q_i \nabla Y_i(z_i^*)^T \hat{\tilde{z}}_i + \dots. \tag{8}$$

where Ξ is an constant.

Substituting the optimal control u^* (35) into the state equation (36), we obtain:

$$\dot{z} = Az + \sum_{i=1}^{N} \left(-B_i R_i^{-1} B_i^T \lambda + \hat{\hat{g}}_i \right). \tag{9}$$

Let $B = [B_1, \cdots, B_N]$, $R = diag\{R_1, \cdot, R_N\}$. Then the above equation can be written as:

$$\dot{z} = Az + -BR^{-1}B^T \lambda + \hat{\hat{g}}. \tag{10}$$

where $\hat{\hat{g}} = \sum_{i=1}^{N} \hat{\hat{g}}_i$.

The costate equation (37) can rewritten as:

$$\dot{\lambda} = -\sum_{i=1}^{N} \frac{\partial}{\partial z} \left(\|Y_i(z_i)\|_{Q_i}^2 \right) - A^T \lambda = -\hat{\Omega}\hat{\hat{z}} - A^T \lambda. \tag{11}$$

where $\hat{\Omega} = \sum_{i=N}^{N} \frac{\partial^2}{\partial z^2} \left(\|Y_i(z_i)\|_{Q_i}^2 \right) |_{z=z^*}$ is the value of the Hessian matrix of the global cost function with respect to the state z, evaluated at the equilibrium point.

Assuming that $\hat{\hat{g}}$ does not vary significantly near the equilibrium point, the linearized coupled system is:

$$\begin{bmatrix} \dot{\hat{\hat{z}}}(t) \\ \dot{\lambda}(t) \end{bmatrix} = \begin{bmatrix} A & -BR^{-1}B^T \\ -\hat{\Omega} & -A^T \end{bmatrix} \begin{bmatrix} \hat{\hat{z}}(t) \\ \lambda(t) \end{bmatrix}, \tag{12}$$

Consider the multi-UAV system with dynamics simplified to single-integrator form under Assumption 1 (slow flight and small attitude angles)[?], then, $A = 0$, $B = I$, for the special case where $R = I_{4N}$ (uniform control penalties), this simplifies to:

$$\begin{gathered} \begin{bmatrix} \dot{z}(t) \\ \dot{\lambda}(t) \end{bmatrix} = \begin{bmatrix} 0 & -I_{8N} \\ -\hat{\Omega} & 0 \end{bmatrix} \begin{bmatrix} z(t) \\ \lambda(t) \end{bmatrix}, \\ \begin{bmatrix} z(0) \\ \lambda(t_f) \end{bmatrix} = \begin{bmatrix} z(0) \\ Fz(t_f) \end{bmatrix}, \end{gathered} \tag{13}$$

Therefore, the matrix $\hat{\Omega}$ is essentially the value of the Hessian matrix of the global cost function J with respect to the state, evaluated at the Nash equilibrium point. It reflects the curvature of the total system cost with respect to changes in the state near the equilibrium. Positive definiteness implies that the cost function is convex in the vicinity of the equilibrium, thereby ensuring that the equilibrium point is locally optimal.
