# Supplementary material for: A distributed differential game approach to trajectory planning for offshore wind farm inspection
Source: PLoS One. 2026 Mar 26;21(3):e0344989. doi: 10.1371/journal.pone.0344989 (PMC13021010; doi:10.1371/journal.pone.0344989)
Supplement: Attachment — Submitted filename: Response to Reviewers.pdf [file pone.0344989.s002.pdf]

Authors' Response to Reviews of

A distributed differential game approach to trajectory planning for offshore wind farm inspection

PLOS ONE Submission: PONE-D-25-54513,

RC: ***Reviewers' Comment***, AR: Authors' Response

Dear Editor

Thank you very much for your recent e-mail concerning the first submission of the paper. We sincerely thank you and the reviewers for the time spent and effort in reviewing this paper. We have studied the comments carefully and have made significant improvements according to the valuable comments and suggestions of the editor and reviewers. We believe after these modifications, the quality of the revised manuscript can meet the requirements of honorary journals. The detailed revisions are marked in red color in the revised paper. Responses to each reviewer's comments are enclosed.

We look forward to hearing from you soon.

Sincerely,

Authors

1. Reviewer #1

1.1. General comments

RC: ***The manuscript presents a solid and technically well-founded proposal by employing a formulation based on the Distributed Differential Game (DDG) framework for trajectory planning of unmanned aerial vehicles (UAVs) engaged in cooperative inspections of offshore wind farms. The study is both relevant and timely, considering the growing application of autonomous systems in maritime environments and the inherent challenges related to communication limitations and energy consumption in multi-agent systems. The text demonstrates an adequate conceptual command of differential game theory and distributed control, establishing a consistent link between trajectory optimization, energy efficiency, and operational safety.***

AR: We would like to express our sincerest appreciation for your constructive comments in the review. The detailed suggestions provided are invaluable for improving the clarity, depth, and practical relevance of our paper. We have carefully considered every comment and have revised the manuscript accordingly. Below, we provide a point-by-point response to each suggestion. All changes in the revised manuscript are highlighted in red for your convenience. We have revised the paper based on your constructive comments. Please find below the detailed response to each of your comments.

1.2. Major concern #1

RC: ***#1-1 - From a scientific standpoint, the paper exhibits clear merit by proposing an integration between optimal control and game theory, resulting in a decentralized approach that overcomes the limitations of traditional centralized methods. The theoretical framework is robust, and the demonstration of convergence from the local Nash equilibrium (L-NE) to the global Nash equilibrium (G-NE) is logically developed. However, the mathematical exposition is dense, and certain sections could benefit from a more didactic presentation—particularly those introducing second-order differential equations and the modelling of the multi-UAV system. A summary table consolidating the variables and parameters used would facilitate readability and enhance the reproducibility of the model.***

AR: Thank you for your comment. We agree that a consolidated list will greatly benefit readers. In the revised manuscript, we have added a new Table 1 in the Preliminaries section. This table lists all major variables, symbols, and parameters used throughout the paper (see the revised paper, pages 3 and 4, for details).

RC: ***#1-2 - The introduction adequately covers the state of the art, but the comparative discussion with deep reinforcement learning (NN-DRL) and genetic optimization (GA-DZ) methods could be expanded to highlight the incremental originality of the proposed approach more clearly.***

AR: Thank you for your comment. We apologize for not having presented our highlight clearly in the first submission. We have enhanced the comparison in two key places:

a) In the Introduction (Pages 2 to 3): We have expanded the discussion to explicitly state:"In summary, while GA-DZ [6] optimizes flight distance (and thus implicitly reduces energy consumption) yet lacks explicit safety and return-trip constraints, and NN-DRL [9] learns adaptive policies but

offers no guarantee of global optimality and is prone to local optima, DMPC [15] can handle dynamic disruptions but often sacrifices inspection efficiency due to its local optimization nature. Furthermore, existing centralized differential game approaches [19, 24] are not directly applicable to offshore wind farm inspection scenarios under communication constraints.

To overcome the above limitations, this paper proposes a novel DDG method, which provides a theoretically guaranteed globally optimal solution. The key contributions are summarized as follows:

1) Compared to the GA-DZ method [6], which requires re-iteration and thus suffers from reduced real-time performance when dealing with dynamic maritime environments, and which optimizes for distance while lacking return-trip constraints, the proposed DDG framework explicitly models round-trip energy constraints and local communication limitations, thereby significantly improving task completion efficiency while ensuring safety. and global convergence.

2) Unlike the NN-DRL approach [9], which learns adaptive strategies yet cannot ensure global optimality and is prone to local optima, the proposed DDG framework provides a theoretically guaranteed convergence from a L-NE to a G-NE for all UAVs. This overcomes the key limitations of learning-based strategies, particularly their lack of theoretical interpretability and convergence assurance.

3) In contrast to the DMPC [15], which relies on iterative algorithmic optimization to obtain locally optimal solutions at each sampling instant, the proposed DDG method is grounded in game theory and explicitly models strategic interactions among UAVs, driving the system toward a NE. Simulations further confirm the superior inspection efficiency of the proposed DDG method over prevailing trajectory planning methods in offshore wind farm applications [6,9,15]."

b) In the Simulation section conclusion (Page 21): "In contrast to GA-DZ's failure in safety and return energy, and NN-DRL's suboptimal time efficiency due to lack of global coordination guarantees, our method ensures both safety and time-optimality by construction through the proven G-NE."

References

[6] Peng Z, Sun S, Tong L, Fan Q, Wang L, Liu D. Optimization of offshore wind farm inspection paths based on K-means-GA. Plos one. 2024;19(5):e0303533.

[9] Fan T, Fu L, Guo C, Zhang Y, Sun L. Multi UAV Inspection Optimization for Offshore Wind Farms Considering Battery Exchange Process. IEEE Transactions on Intelligent Vehicles. 2024.

[15] Kong S, Liu Y. Model predictive control for risk-based inspection and maintenance planning of offshore wind turbines. Ships and Offshore Structures. 2025:1-12.

[19] Cappello D, Garcin S, Mao Z, Sassano M, Paranjape A, Mylvaganam T. A hybrid controller for multi-agent collision avoidance via a differential game formulation. IEEE Transactions on Control Systems Technology. 2020;29(4):1750-7.

[24] Wang X, Xiao Z, Ren Z, Dong C, Tian XD. Formation cooperative trajectory tracking control for unmanned aerial vehicles via differential game and reinforcement learning. Transactions of the Institute of Measurement and Control. 2025;47(9):1762-70.

RC: ***#1-3 - The simulations are relevant and effectively illustrate trajectory behaviour, although figure captions could be refined for greater interpretive clarity.***

AR: Thank you. We have thoroughly revised all figure captions (Figures 1, 2, 3, 4, 5) to make them

more descriptive and self-contained. Each caption now clearly states (a) what is being shown, (b) the key elements in the figure, and (c) the main takeaway relevant to the narrative. For example, the caption for Figure 3 now reads(Page 19): "Fig 3. Comparison of inspection trajectories for 3 UAVs and 28 turbines: (a) Proposed DDG method: All UAVs (UAV1-3) complete inspection tasks with coordinated, collision-free paths and return to the OBS. (b) GA-DZ method [6]: UAV1 collides with Wind Turbine 4 (marked by 'X'), and UAV3 exhausts its battery before returning. (c) NN-DRL method [9]: All tasks are completed safely, but UAV1 exhibits an inefficient, elongated path requiring mid-mission returns to the OBS, increasing total time.

RC: ***#1-4 - It would also be desirable to include a more detailed analysis of the computational cost of the proposed solution and an assessment of the model's scalability as the number of agents increases—an essential aspect in distributed systems.***

AR: Thank you for your comment. We apologize for not having presented our results clearly in the first submission. To further show the computational cost of the proposed solution and an assessment of the model's scalability as the number of agents increases—an essential aspect in distributed systems, we have supplemented the following simulation results and added in the revised paper, page 21.

"To demonstrate the scalability and effectiveness of the proposed method, a scenario with six UAVs inspecting 40 wind turbines is designed. UAV1 is assigned turbines $\{1, 7, 13, 19, 25, 31, 37\}$; UAV2: $\{2, 8, 14, 20, 26, 32, 38\}$; UAV3: $\{3, 9, 15, 21, 27, 33, 39\}$; UAV4: $\{4, 10, 16, 22, 28, 34, 40\}$; UAV5:$\{5, 11, 17, 23, 29, 35\}$; and UAV6: $\{6, 12, 18, 24, 30, 36\}$. Figure 6 shows the inspection trajectories for the three methods, with sequences summarized in a Table 5(see the revised paper, in the page 22). The proposed method again achieves the shortest total path length (272+226+206+312+240+308=1564 m). UAV1 returns to recharge when its battery drops below a threshold and then resumes inspection. With the GA-DZ method [6], UAV1 collides with turbine 16 while flying from turbine 7 to the next target, preventing further inspection (Figure 6(b), red solid line). UAV3 fails to complete its task due to insufficient battery (Figure 6(b), blue dashed line). The NN-DRL method [9] produces the longest total path length, with UAV1 and UAV4 each returning to charge twice, significantly increasing the path length (Figure 6(c), red and yellow solid lines)."

Similarly, Figure 7 plots the minimum distances between each UAV and obstacles. Figure 7(a) shows that all UAVs maintain safe distances with the proposed method. Figure 7(b) illustrates that for the GA-DZ method [6], UAV1 violates the safe distance, and UAV3 stops at 180 s due to battery depletion. Figure 7(c) shows that all UAVs maintain safe distances with the NN-DRL method [9].

Figure 8 compares the state error convergence for the six-UAV scenario. Figure 8(a) shows that all UAVs' state errors converge to zero with the proposed method, indicating successful task completion. Figure 8(b) reveals that for the GA-DZ method [6], the state errors of UAV1 and UAV3 do not converge to zero, meaning these UAVs fail to complete their inspections. Figure 8(c) shows that all UAVs' state errors converge to zero with the NN-DRL method [9], confirming task completion.

To further illustrate the computational cost of the proposed method, the comparison results of online average computation time for the three methods under different paradigms are shown in Figure 9. In the scenario of 3 UAVs inspecting 28 wind turbines, the online average computation time of the proposed DDG method is 0.15 seconds, which outperforms GA-DZ [6] at 0.50 seconds and NN-DRL [9] at 0.30 seconds. The GA-DZ method suffers from significant computational burden due to its reliance on online genetic algorithm optimization. The NN-DRL method, while better than GA-DZ, still requires periodic online learning updates, resulting in higher computation times. When scaling up to 6 UAVs inspecting 40 wind turbines, the computation time of the

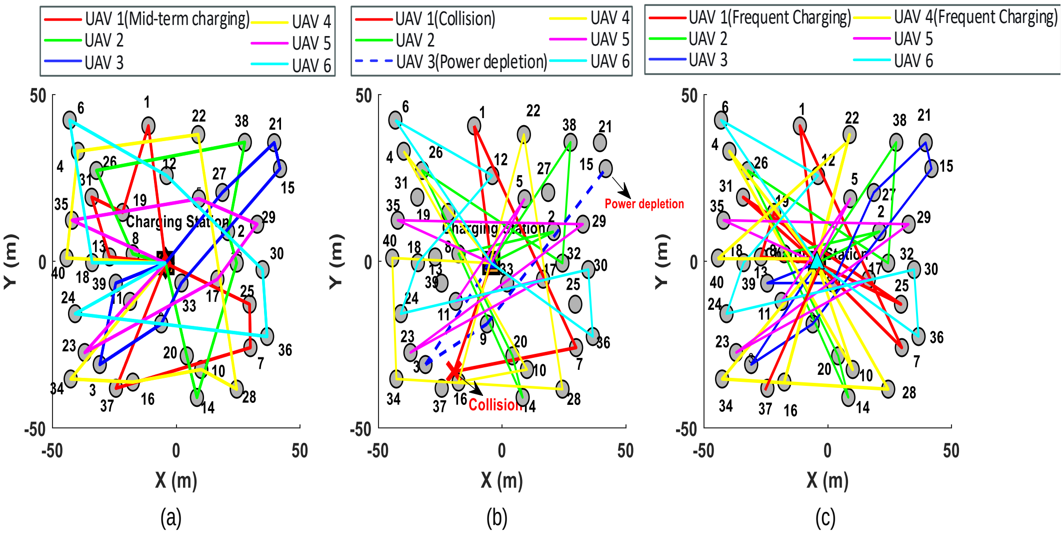

Figure 1: Comparison of inspection trajectories for 6 UAVs and 40 turbines: (a) Proposed DDG method: All UAVs (UAV1-6) complete inspection tasks on coordinated, collision-free paths and return to the OBS, though UAV1's elongated path requires it to return to the OBS mid-mission. (b) GA-DZ method [6]: UAV1 collides with Wind Turbine 16 (marked by 'X'), and UAV3 exhausts its battery before returning. (c) NN-DRL method [9]: All tasks are completed safely, but UAV1 and UAV4 exhibits an inefficient, elongated path requiring mid-mission returns to the OBS, increasing total time.

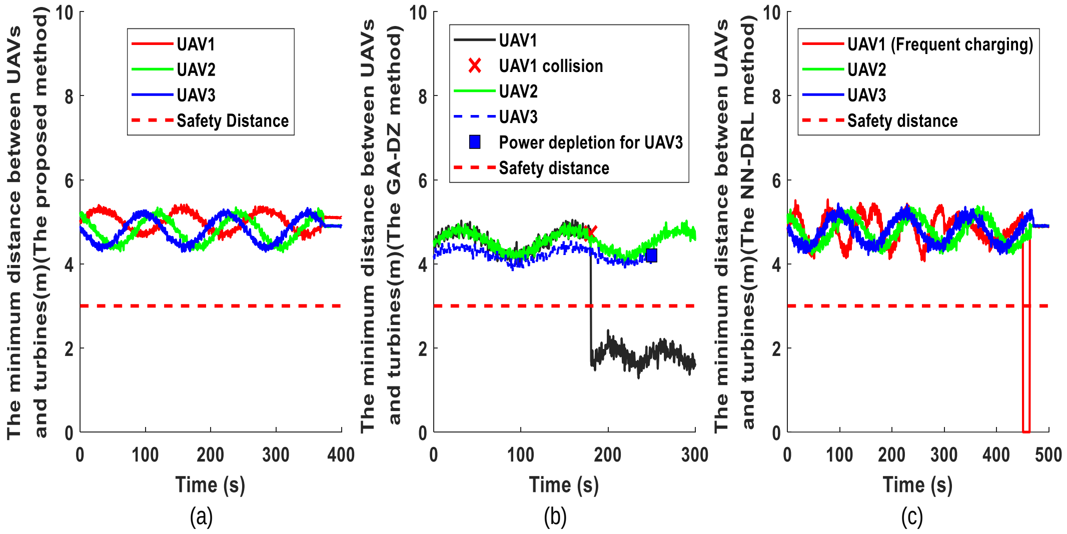

Figure 2: 6 UAVs and 40 turbines:(a)The minimum distance between UAVs and wind turbines with the proposed method (b)The minimum distance between UAVs and wind turbines with the GA-DZ method (c)The minimum distance between UAVs and wind turbines with the NN-DRL method.

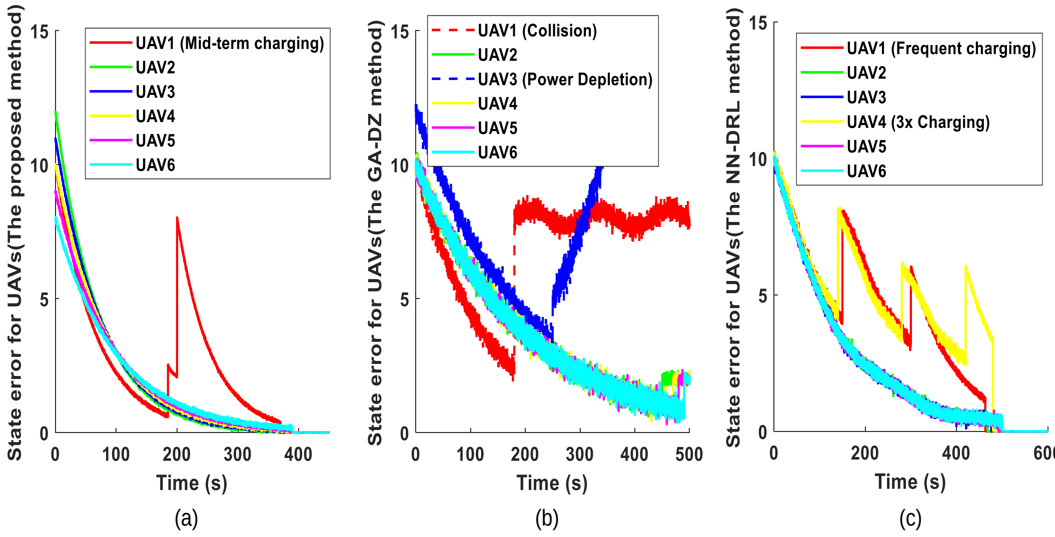

Figure 3: The state errors for 6 UAVs and 40 turbines:(a) under proposed approach (b) under the GA-DZ approach (c) under the NN-DRL approach.

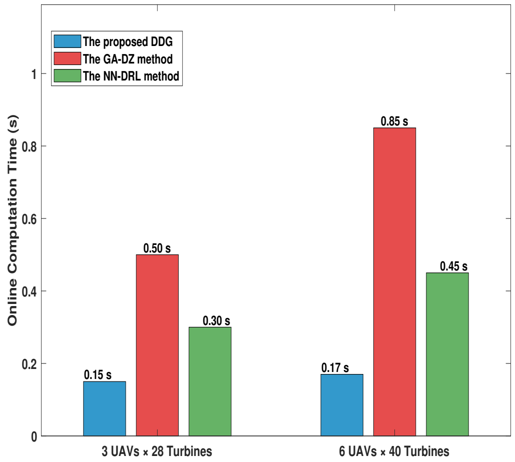

Figure 4: Comparison of the online average computation time for the three methods under two scales.

DDG method only increases to 0.17 seconds, a growth rate of 13.3%, significantly lower than the 70.0% of GA-DZ and 50.0% of NN-DRL. This notable advantage stems from the core design of the distributed differential game framework: each UAV only needs to solve a local optimal control problem (Eq. 18-20), whose computational complexity depends solely on its own state and the number of neighbors $\mathcal{N}_i$, independent of the total system scale N, thereby ensuring the scalability of individual computations. At the same time, the distributed nature of the algorithm leads to a near-linear increase in the total system-wide computational load with respect to N, avoiding the combinatorial explosion or polynomial complexity often encountered in centralized global optimizers; the communication overhead scales with the network density (number of edges in graph $G(\mathcal{V}, \varepsilon)$), and under the assumption of a strongly connected graph, the system is guaranteed to converge to a G-NE regardless of how N increases. By incorporating a local information interaction mechanism, the proposed method achieves a gradual increase in computational burden with scale, making it more suitable for real-world offshore wind farm inspection scenarios characterized by limited communication and variable scales. "

To enhance the presentation, in the paper (pages 25 to 26; Scalability Analysis) we added the Scalability Analysis subsection in the Simulations Section" To concretely address scalability and computational burden, we performed a head-to-head comparison between our distributed DDG and an equivalent centralized game-theoretic solver. The centralized solver uses the same PMP principle and the proposed cost structure but optimizes the trajectories of all UAVs simultaneously using global information. The results are presented in the Figure 5(Figure 11 in the revised paper).

1) For the 3-UAV / 28-turbine scenario

The proposed DDG: Per-UAV computation times are 0.12s, 0.15s, and 0.13s (average: $0.133s$).

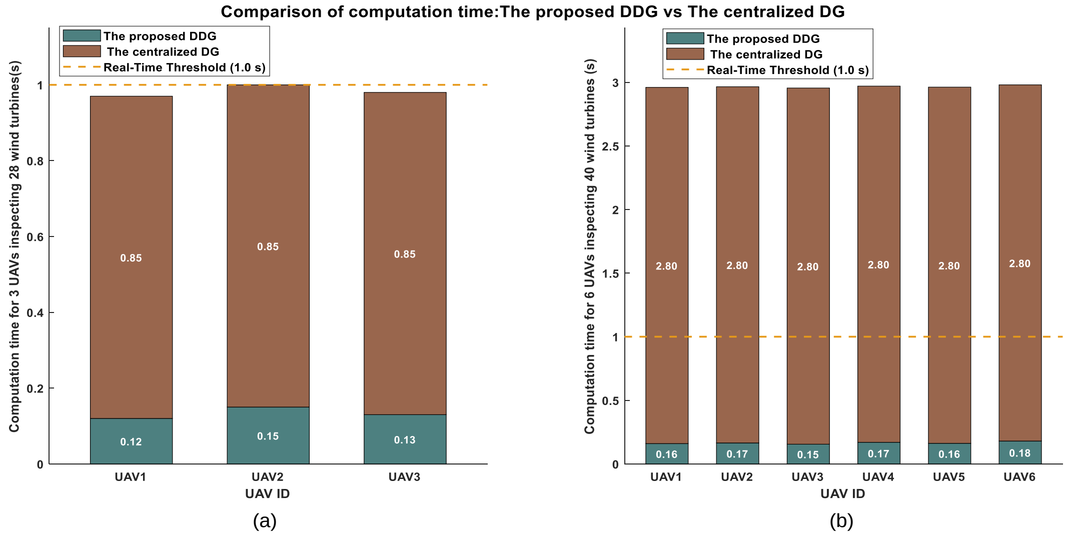

Figure 5: (a) The comparison of computation time for 3 UAVs inspecting 28 wind turbines (b) The comparison of computation time for 6 UAVs inspecting 40 wind turbines.

The centralized solver: The joint optimization requires 0.85s to compute a solution for the entire system. Therefore, the distributed system is 6.5 times faster per planning cycle when considering parallel execution.

2) For the 6-UAV / 40-turbine scenario:

The proposed DDG: Per-UAV times range from 0.155s to 0.18s (average: $0.165s$).

The centralized solver: Computation time surges to $2.8s$.

Then, the performance gap widens significantly. The distributed system is now 17 times faster on average, and critically, the centralized time far exceeds the $1.0s$ real-time threshold. This comparative analysis provides direct, empirical evidence that our proposed DDG framework successfully avoids the combinatorial explosion typical of centralized optimal control.

Notably, when the fleet size was increased by 100% (from 3 to 6 UAVs), the average per-agent computation time of our DDG method increased by only about 24%. This sub-linear growth is a direct outcome of the distributed architecture, where each UAV solves a local problem whose complexity is bounded by the size of its neighborhood $|\mathcal{N}_i|$, rather than by the global fleet size N."

Furthermore, We have added a new Remark (The revised paper, page 12)" **Remark 6** To illustrate the scalability of the proposed DDG method, an analysis is conducted from two aspects: computational burden and real-time feasibility.

1) Computational burden: Solving the local optimal control problem (Eq. 18-20) for each UAV involves a two-point boundary value problem with state dimension 8. We employ an efficient iterative solver (i.e., a gradient-based method) whose convergence per agent typically requires $\mathcal{O}(10^2)$ iterations in our simulations, with each iteration involving low-dimensional matrix operations [16].

The distributed architecture allows these computations to be parallelized across UAVs.

2) Real-time feasibility: For the inspection scenarios considered (mission duration 400s), the trajectory planning is computed offline or re-planned at low frequency (every 30s) based on updated neighbor states. The per-agent computation time (0.5s on a standard desktop CPU) is negligible compared to the re-planning interval, demonstrating the method's potential for near real-time operation.

We also compare our proposed approach with a centralized game-theoretic solver, which employs the same PMP principle and cost structure as our DDG method but solves a single, high-dimensional optimization problem using global information (i.e., centralized DG). This centralized solver must handle the concatenated state vector of all N UAVs, resulting in a total state dimension of $8N$. Consequently, its computational complexity scales approximately as $\mathcal{O}((N \times n)^3)$, where $n = 8$ represents the state dimension of a single UAV."

References

[16] Y. Li and X. Hu, A differential game approach to intrinsic formation control, Automatica 136 (2022) p.110077.

RC: ***#1-5 - From a theoretical perspective, the proposed formulation is consistent and aligns with the principles of multi-objective optimization applied to cooperative dynamic systems. The demonstration of convergence toward the G-NE, based on the positive definiteness of the $\hat{\Omega}$ matrix, is well-structured, but would benefit from a more rigorous explanation of the relationship between communication graph connectivity and the uniqueness of the solution.***

AR: We thank the reviewer for highlighting this crucial theoretical aspect. The strong connectivity of graph $G(\mathcal{V}, \varepsilon)$ ensures that information (and thus strategy influences) can propagate between any two UAVs in the network. This global propagative property, combined with the positive definiteness of $\hat{\Omega}$ which guarantees the uniqueness of the solution to the adjoint system (33), prevents the existence of isolated or conflicting local equilibria. Consequently, the locally consistent strategy u_i^* derived from neighborhood interactions is forced to be consistent across the entire network, leading to a unique system-wide equilibrium (G-NE).

This clarifies that strong connectivity is the topological condition that, when coupled with the analytical condition of positive definite $\hat{\Omega}$, ensures the local solutions stitch together into a unique global solution.

We have expanded the proof and discussion of Proposition 1 (Pages 13 to 16) to explicitly articulate this link, we also added Remark 7(Page 16) :" **Remark 7** The strong connectivity of the communication graph $G(\mathcal{V}, \varepsilon)$ ensures a bidirectional information path between any two UAVs, allowing local strategy information to propagate across the entire network in finite time. This leads to global alignment of the L-NE strategies and consequently drives convergence toward a G-NE. Furthermore, the positive definiteness of $\hat{\Omega}$ guarantees the existence and uniqueness of the L-NE, which in turn ensures that the resulting G-NE is also uniquely defined. "

RC: ***#1-6 - Similarly, practical aspects such as communication delays, wind variation, and possible link losses could be discussed as limitations or directions for future work, which would add both realism and practical depth to the proposed model.***

AR: Thank you for your comment. We agree that discussing these practical factors adds important realism and depth. We have added a new subsection titled "Limitations and Future Work" just before the Conclusion (Pages 28 to 29):"

Current Limitations: The present model assumes ideal, delay-free communication within the sensing range and does not explicitly account for dynamic environmental disturbances such as wind gusts.

To enhance the realism and robustness of the proposed framework, future work will focus on incorporating time-varying communication topologies, developing communication delay compensation mechanisms, modeling dynamic wind fields, and formulating strategies to handle unexpected obstacles. Integrating stochastic wind models into the dynamics and cost function for more resilient trajectory planning. Additionally, large-scale simulations (e.g., $N > 20$) will be conducted to empirically quantify the relationship between system scale and performance, further validating the scalability and practical applicability of the method."

2. Reviewer #2

2.1. General comments

RC: ***The manuscript introduces a distributed differential game (DDG) framework for optimal trajectory planning of multiple UAVs inspecting offshore wind farms. It addresses critical issues like energy constraints, local communication, and round-trip guarantees. The theoretical analysis includes convergence to global Nash equilibrium (G-NE), supported by simulations comparing the proposed method against GA-DZ and NN-DRL baselines.***

AR: The authors would like to thank the reviewer for recoganizing the main contribution of this paper. Following your constructive comments, we have duly revised the paper. The response to your comments are detailed in the follows.

2.2. Major concern #2

RC: ***#2-1 - The GA-DZ and NN-DRL benchmarks lack implementation details. There is no mention of training setup, DRL architecture, or hyperparameter tuning. Fairness of comparison is unclear.***

AR: We thank the reviewer for this critical point. To ensure a fair and reproducible comparison, we have added the implementation details of benchmark methods in the Simulations section (Pages 16 to 17)"For the GA-DZ baseline, we adopted the genetic algorithm with dynamic zoning as outlined in Ref.[6], utilizing the authors' publicly available source code. The algorithm encodes solutions as sequences of waypoint assignments and corresponding flight trajectories. Its optimization is driven by a fitness function defined as the inverse of the total path distance for the UAV fleet. To ensure a meaningful comparison that tests the algorithm's inherent ability to satisfy constraints, substantial penalties (1×10^6 per violation) are applied in the fitness evaluation for any path that violates safety distances limits. The evolutionary process uses a population size of 100 and runs for 500 generations per simulation, with a crossover rate of 0.85, a mutation rate of 0.1, and tournament selection (size=3). Unlike learning-based methods, GA-DZ does not involve a separate training phase; it is executed directly on each test scenario to produce a scenario-specific solution, enabling a direct and fair performance comparison under the same conditions as the proposed DDG method.

For the NN-DRL baseline, we implemented a Dueling Deep Q-Network (DQN) following the architecture in Ref.[9]. The network takes the UAV's state as input, processes it through two fully-connected layers, and outputs Q-values for each discrete action. The state space includes the UAV's pose p_i, velocity v_i, remaining battery, relative positions to its target and the nearest wind turbine, as well as positions of neighboring UAVs within communication range. The action space is defined as nine discrete actions: hovering and moving in eight fixed-speed directions. The reward function is designed to balance multiple objectives and is formulated as:

Table 1: The parameters of the NN-DRL method [9].

Parameter	Value
Learning rate	0.001
Discount factor	0.99
Replay buffer size	50000
Batch size	64
Target update frequency	100*step*
Total number of training episodes	10000

We consider a multi-UAV inspection system comprising three UAVs (i.e., $N = 3$). The environment contains 28 offshore wind turbines and one offshore booster station (OBS), which serves as both the charging base and the common starting point for all UAVs. The simulated obstacles include known wind turbine towers, each with a radius of $5m$, as well as other UAVs, which are treated as unknown dynamic obstacles. Each UAV is assigned a specific subset of wind turbines for inspection and plans its trajectory accordingly. To ensure a fair comparison, the proposed DDG method and the two benchmark approaches [6, 9] are evaluated under identical conditions: the same initial positions and dynamics model (Eq. 3), the same environmental layout (28 turbines and 1 OBS), and the same success criteria (complete inspection, collision-free operation, and safe return to the OBS). This setup isolates the performance differences to the algorithmic level."

References

[6] Peng Z, Sun S, Tong L, Fan Q, Wang L, Liu D. Optimization of offshore wind farm inspection paths based on K-means-GA. Plos one. 2024;19(5):e0303533.

[9] Fan T, Fu L, Guo C, Zhang Y, Sun L. Multi UAV Inspection Optimization for Offshore Wind Farms Considering Battery Exchange Process. IEEE Transactions on Intelligent Vehicles. 2024.

RC: ***#2-2 - The system is only tested on 3 UAVs. There is no analysis on how the framework performs with larger teams, which is critical for offshore inspection scalability..***

AR: Thank you for your raising this concern. We understand the your concern that the information with only three UAVs is insufficient to demonstrate the effectiveness of the proposed method. To strengthen our results, six UAVs are introduced to validate the practicality of the proposed method. To enhance the presentation, in the paper (pages 20 to 23) we added "To demonstrate the scalability and effectiveness of the proposed method, a scenario with six UAVs inspecting 40 wind turbines is designed. UAV1 is assigned turbines $\{1, 7, 13, 19, 25, 31, 37\}$; UAV2: $\{2, 8, 14, 20, 26, 32, 38\}$; UAV3: $\{3, 9, 15, 21, 27, 33, 39\}$; UAV4: $\{4, 10, 16, 22, 28, 34, 40\}$; UAV5:$\{5, 11, 17, 23, 29, 35\}$; and UAV6: $\{6, 12, 18, 24, 30, 36\}$. Figure 6 shows the inspection trajectories for the three methods, with sequences summarized in a Table 5. The proposed method again achieves the shortest total path length (272+226+206+312+240+308=1564 m). UAV1 returns to recharge when its battery drops below a threshold and then resumes inspection. With the GA-DZ method [6], UAV1 collides with turbine 16 while flying from turbine 7 to the next target, preventing further inspection (Figure 6(b), red solid line). UAV3 fails to complete its task due to insufficient battery (Figure 6(b), blue dashed

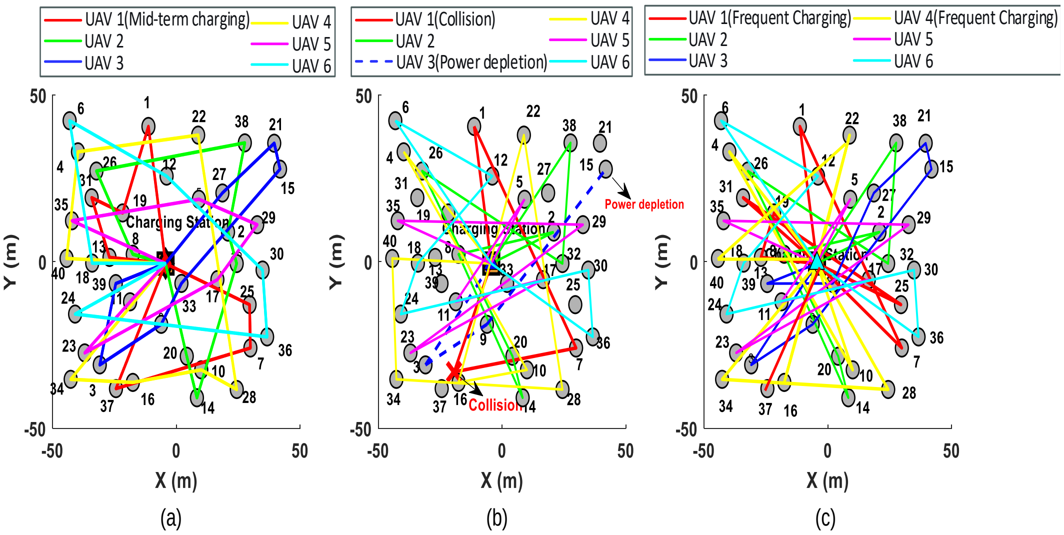

Figure 6: Comparison of inspection trajectories for 6 UAVs and 40 turbines: (a) Proposed DDG method: All UAVs (UAV1-6) complete inspection tasks on coordinated, collision-free paths and return to the OBS, though UAV1's elongated path requires it to return to the OBS mid-mission. (b) GA-DZ method [6]: UAV1 collides with Wind Turbine 16 (marked by 'X'), and UAV3 exhausts its battery before returning. (c) NN-DRL method [9]: All tasks are completed safely, but UAV1 and UAV4 exhibits an inefficient, elongated path requiring mid-mission returns to the OBS, increasing total time.

line). The NN-DRL method [9] produces the longest total path length, with UAV1 and UAV4 each returning to charge twice, significantly increasing the path length (Figure 6(c), red solid line).

Table 2: The inspection sequences and path length with the three methods.

Method	Inspection sequences
The proposed method	UAV1:$\{13 \rightarrow 31 \rightarrow 19 \rightarrow 1 \rightarrow Charging \rightarrow 25 \rightarrow 7 \rightarrow 37\}(272m)$
	UAV2: $\{8 \rightarrow 26 \rightarrow 38 \rightarrow 2 \rightarrow 32 \rightarrow 14 \rightarrow 20\}(226m)$
	UAV3: $\{39 \rightarrow 3 \rightarrow 9 \rightarrow 33 \rightarrow 15 \rightarrow 21 \rightarrow 27\}$ $(206m)$
	UAV4: $\{40 \rightarrow 4 \rightarrow 22 \rightarrow 28 \rightarrow 10 \rightarrow 16 \rightarrow 34\}$ $(312m)$
	UAV5: $\{11 \rightarrow 23 \rightarrow 17 \rightarrow 29 \rightarrow 5 \rightarrow 35\}$ $(240m)$
	UAV6: $\{18 \rightarrow 6 \rightarrow 12 \rightarrow 30 \rightarrow 36 \rightarrow 24\}$ $(308m)$
The GA-DZ method	UAV1: $\{1 \rightarrow 7\}$(Collision)$(208m)$
	UAV2: $\{38 \rightarrow 32 \rightarrow 26 \rightarrow 20 \rightarrow 14 \rightarrow 8 \rightarrow 2\}(235m)$
	UAV3: $\{3 \rightarrow 9 \rightarrow 15\}$ (Power depletion)$(135m)$
	UAV4: $\{22 \rightarrow 28 \rightarrow 16 \rightarrow 34 \rightarrow 40 \rightarrow 4 \rightarrow 10\}$ $(450m)$
	UAV5: $\{35 \rightarrow 29 \rightarrow 23 \rightarrow 17 \rightarrow 11 \rightarrow 5\}(353m)$
	UAV6: $\{6 \rightarrow 12 \rightarrow 18 \rightarrow 24 \rightarrow 30 \rightarrow 36\}$ $(323m)$
The NN-DRL method	UAV1: $\{1 \rightarrow 7 \rightarrow Charging \rightarrow 31 \rightarrow 25 \rightarrow 13 \rightarrow 19 \rightarrow Charging \rightarrow 37\}$ $(366m)$
	UAV2: $\{38 \rightarrow 32 \rightarrow 26 \rightarrow 20 \rightarrow 14 \rightarrow 8 \rightarrow 2\}(235m)$
	UAV3: $\{39 \rightarrow 33 \rightarrow 27 \rightarrow 21 \rightarrow 15 \rightarrow 9 \rightarrow 3\}$ $(248m)$
	UAV4: $\{28 \rightarrow 16 \rightarrow 34 \rightarrow Charging \rightarrow 4 \rightarrow 10 \rightarrow 40 \rightarrow Charging \rightarrow 22\}$ $(398m)$
	UAV5: $\{35 \rightarrow 29 \rightarrow 23 \rightarrow 17 \rightarrow 11 \rightarrow 5\}(353m)$
	UAV6: $\{6 \rightarrow 12 \rightarrow 18 \rightarrow 24 \rightarrow 30 \rightarrow 36\}(323m)$

Similarly, Figure 7 plots the minimum distances between each UAV and obstacles. Figure 7(a) shows that all UAVs maintain safe distances with the proposed method. Figure 7(b) illustrates that for the GA-DZ method [6], UAV1 violates the safe distance, and UAV3 stops at 180 s due to battery depletion. Figure 7(c) shows that all UAVs maintain safe distances with the NN-DRL method [9].

Figure 8 compares the state error convergence for the six-UAV scenario. Figure 8(a) shows that all UAVs' state errors converge to zero with the proposed method, indicating successful task completion. Figure 8(b) reveals that for the GA-DZ method [6], the state errors of UAV1 and UAV3 do not converge to zero, meaning these UAVs fail to complete their inspections. Figure 8(c) shows that all UAVs' state errors converge to zero with the NN-DRL method [9], confirming task completion.

In contrast to GA-DZ's failure in safety and return energy, and NN-DRL's suboptimal time efficiency due to lack of global coordination guarantees, our method ensures both safety and time-optimality by construction through the proven G-NE."

To further illustrate the computational cost of the proposed method, the comparison results of

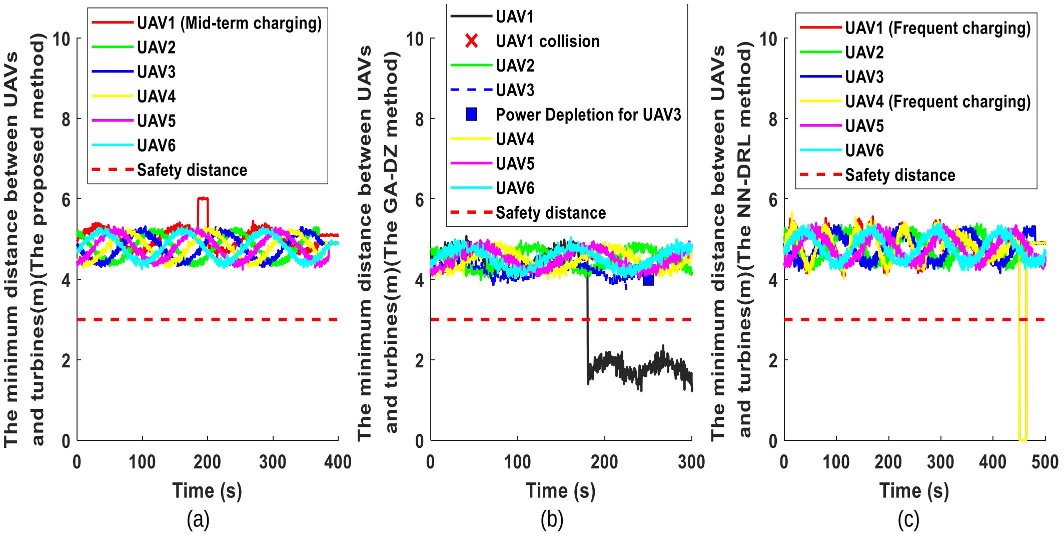

Figure 7: 6 UAVs and 40 turbines:(a)The minimum distance between UAVs and wind turbines with the proposed method (b)The minimum distance between UAVs and wind turbines with the GA-DZ method (c)The minimum distance between UAVs and wind turbines with the NN-DRL method.

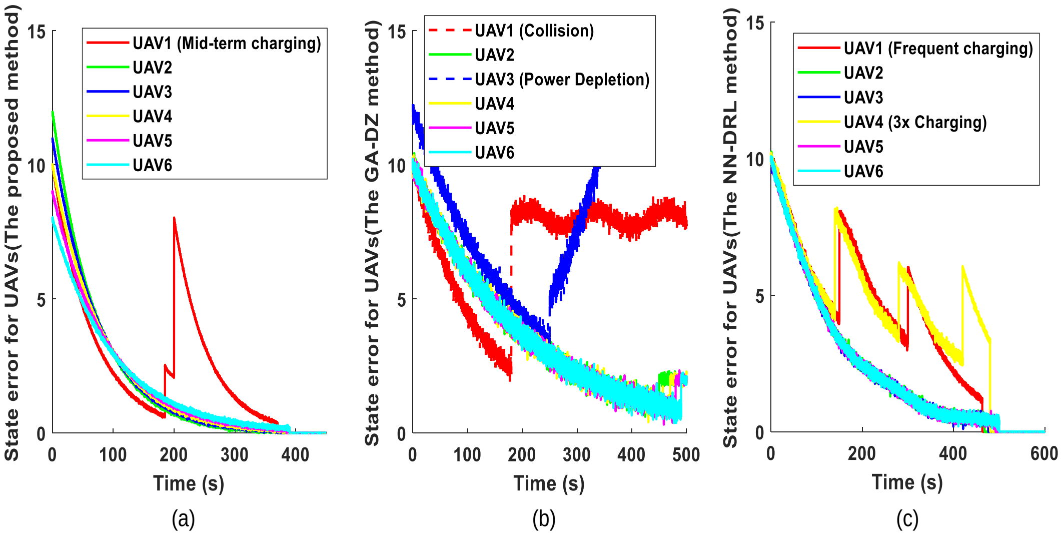

Figure 8: The state errors for 6 UAVs and 40 turbines:(a) under proposed approach (b) under the GA-DZ approach (c) under the NN-DRL approach.

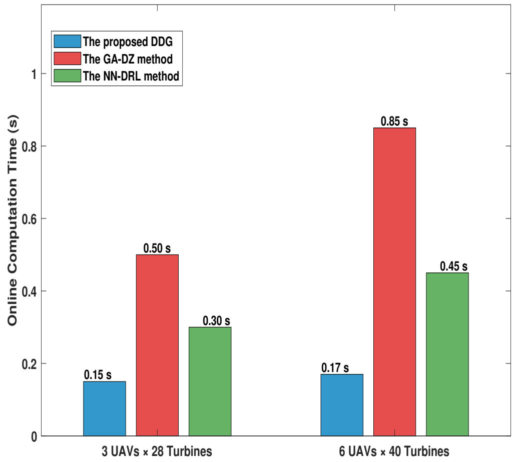

Figure 9: Comparison of the online average computation time for the three methods under two scales.

online average computation time for the three methods under different paradigms are shown in Figure 9. In the scenario of 3 UAVs inspecting 28 wind turbines, the online average computation time of the proposed DDG method is 0.15 seconds, which outperforms GA-DZ [6] at 0.50 seconds and NN-DRL [9] at 0.30 seconds. The GA-DZ method suffers from significant computational burden due to its reliance on online genetic algorithm optimization. The NN-DRL method, while better than GA-DZ, still requires periodic online learning updates, resulting in higher computation times. When scaling up to 6 UAVs inspecting 40 wind turbines, the computation time of the DDG method only increases to 0.17 seconds, a growth rate of 13.3%, significantly lower than the 70.0% of GA-DZ and 50.0% of NN-DRL. This notable advantage stems from the core design of the distributed differential game framework: each UAV only needs to solve a local optimal control problem (Eq. 18-20), whose computational complexity depends solely on its own state and the number of neighbors $\mathcal{N}_i$, independent of the total system scale N, thereby ensuring the scalability of individual computations. At the same time, the distributed nature of the algorithm leads to a near-linear increase in the total system-wide computational load with respect to N, avoiding the combinatorial explosion or polynomial complexity often encountered in centralized global optimizers; the communication overhead scales with the network density (number of edges in graph $G(\mathcal{V}, \varepsilon)$), and under the assumption of a strongly connected graph, the system is guaranteed to converge to a G-NE regardless of how N increases. By incorporating a local information interaction mechanism, the proposed method achieves a gradual increase in computational burden with scale, making it more suitable for real-world offshore wind farm inspection scenarios characterized by limited communication and variable scales. "

RC: ***#2-3 -The convergence from L-NE to G-NE assumes a positive definite matrix $\hat{\Omega}$ but does not explain how it is constructed or verified in practice. This weakens the rigor of the theoretical claim.***

AR: We thank the reviewer for highlighting this gap in the theoretical exposition. We have revised the Proof of Proposition 1 (Pages 14 to 15) to clarify:"To establish the uniqueness of the G-NE, we begin by analyzing the adjoint system derived from a quadratic approximation of the problem around the equilibrium trajectory. Consider the Nash equilibrium trajectory z^*, Defining the deviation as $\hat{\tilde{z}} = z - z^*$, we have the collective state vector $\hat{\tilde{z}} = [\hat{\tilde{z}}_1^T, \cdots, \hat{\tilde{z}}_N^T]^T \in \mathbb{R}^{8N}$ and the co-state vector $\lambda = [\lambda_1^T, \cdots, \lambda_N^T]^T \in \mathbb{R}^{8N}$. The associated adjoint system with two-point boundary values is given by (The derivation is detailed in Appendix A):

$$\begin{aligned} \begin{bmatrix} \dot{\hat{\tilde{z}}}(t) \\ \dot{\lambda}(t) \end{bmatrix} &= \begin{bmatrix} 0 & -I_{8N} \\ -\hat{\Omega} & 0 \end{bmatrix} \begin{bmatrix} \hat{\tilde{z}}(t) \\ \lambda(t) \end{bmatrix}, \\ \begin{bmatrix} \hat{\tilde{z}}(0) \\ \lambda(t_f) \end{bmatrix} &= \begin{bmatrix} \hat{\tilde{z}}(0) \\ Fz(t_f) \end{bmatrix}, \end{aligned} \tag{1}$$

where the matrix $\hat{\Omega} \in \mathbb{R}^{8N \times 8N}$ is a block-diagonal matrix, $\hat{\Omega} = diag\{\hat{\Omega}_1, \cdots, \hat{\Omega}_i, \cdots, \hat{\Omega}_N\}$. Each block is constructed from the Hessian of the running cost function for UAV i evaluated at the equilibrium:

$$\hat{\Omega} = \sum_{i=1}^{N} \alpha_i \nabla^2 Y_i(z_i^*). \tag{2}$$

where z_i^* is the state of UAV i at the Nash equilibrium, $\nabla^2 Y_i(z_i^*)$ is the Hessian matrix of Y_i at z_i^*, $\alpha_i > 0$ is a positive weighting coefficient. The aggregate matrix $\hat{\Omega}$ thus represents a weighted sum of the individual Hessians.

The positive definiteness of $\hat{\Omega}$ is crucial and follows from the construction of the running cost $Y_i(z_i)$. This cost combines a collision avoidance penalty $D(z_i)$ and a trajectory optimization term $DS(z_i(t))$. The penalty term $D(z_i)$ is designed to be convex and increasing outside a safe distance, with a positive definite Hessian at the collision-free equilibrium point z_i^*. The trajectory term $DS(z_i(t))$ is also convex (e.g., based on squared angular deviation), yielding a positive semi-definite Hessian. By selecting positive coefficients α_i, the weighted superposition of these terms ensures that each $\nabla^2 Y_i(z_i^*)$ is positive semi-definite, with at least one block being positive definite. Consequently, the block-diagonal matrix $\hat{\Omega}$ is positive definite.

The positive definiteness of $\hat{\Omega}$ together with the positive definiteness of the weighting matrices Q_i, R_i, R_{ij}, F_i in the cost function (9), guarantees that the integrated cost term $\|Y_i(z_i)\|_{Q_i}^2 + \|u_i(t)\|_{R_i}^2 + \sum_{j \in \mathcal{N}_i} \|u_{ij}\|_{R_{ij}}^2$ and the terminal cost $\psi(z_i(t))$ are jointly convex. Therefore, given the strategies of its neighbors u_{-i}^*, each UAV's optimization problem is strictly convex. For a strictly convex problem, any point satisfying the first-order necessary condition (i.e., the zero-gradient condition) is the unique global minimizer. Thus, the strategy u_i^* obtained in Step 1 is the unique optimal response of UAV i to u_{-i}^*, each UAV's optimization subproblem is strictly convex. For a strictly convex problem, any point satisfying the first-order necessary optimality conditions (i.e., the zero-gradient condition derived from Pontryagin's Minimum Principle) is the unique global minimizer. Hence, the strategy u_i^* obtained upon convergence of the distributed gradient algorithm is the unique optimal response of UAV i to its neighbors' strategies $u_{\hat{i}}^*$."

RC: ***#2-4 -The method involves solving a two-point boundary value problem for each UAV, but computational complexity or real-time feasibility is not discussed.***

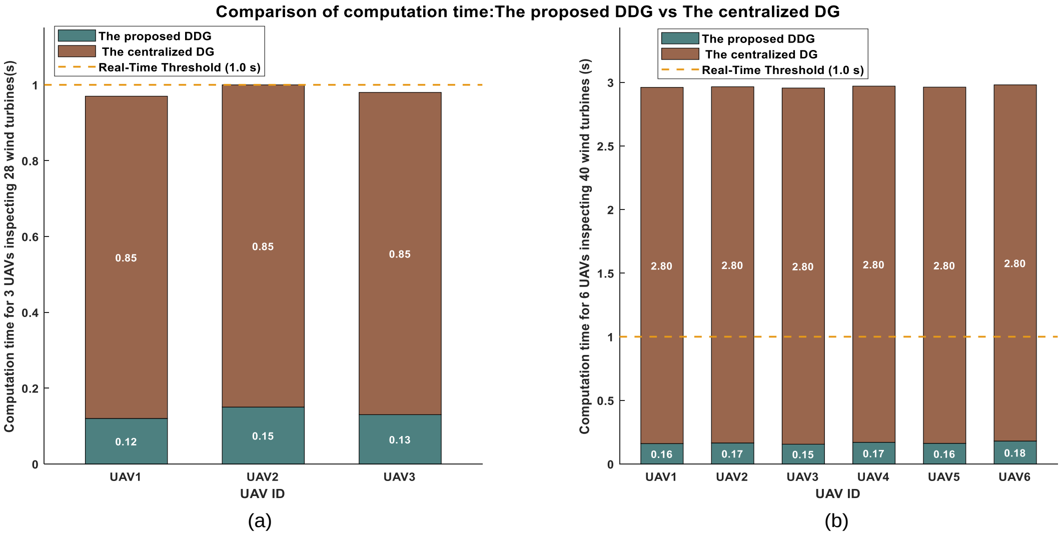

Figure 10: (a) The comparison of computation time for 3 UAVs inspecting 28 wind turbines (b) The comparison of computation time for 6 UAVs inspecting 40 wind turbines.

AR: Thank you for your raising this concern. We apologize for not having presented our results clearly in the first submission. we added a new subsection "Scalability Analysis" (Pages 25 to 26, Scalability Analysis subsection)"To concretely address scalability and computational burden, we performed a head-to-head comparison between our distributed DDG and an equivalent centralized game-theoretic solver. The centralized solver uses the same PMP principle and the proposed cost structure but optimizes the trajectories of all UAVs simultaneously using global information. The results are presented in the Figure 11.

1) For the 3-UAV / 28-turbine scenario

The proposed DDG: Per-UAV computation times are 0.12s, 0.15s, and 0.13s (average: $0.133s$).

The centralized solver: The joint optimization requires 0.85s to compute a solution for the entire system. Therefore, the distributed system is 6.5 times faster per planning cycle when considering parallel execution.

2) For the 6-UAV / 40-turbine scenario:

The proposed DDG: Per-UAV times range from 0.155s to 0.18s (average: $0.165s$).

The centralized solver: Computation time surges to $2.8s$.

Then, the performance gap widens significantly. The distributed system is now 17 times faster on average, and critically, the centralized time far exceeds the $1.0s$ real-time threshold. This comparative analysis provides direct, empirical evidence that our proposed DDG framework successfully avoids the combinatorial explosion typical of centralized optimal control.

Notably, when the fleet size was increased by 100% (from 3 to 6 UAVs), the average per-agent

computation time of our DDG method increased by only about 24%. This sub-linear growth is a direct outcome of the distributed architecture, where each UAV solves a local problem whose complexity is bounded by the size of its neighborhood $|\mathcal{N}_i|$, rather than by the global fleet size N."

To enhance our presentation, in the paper (page 12; Remark 6.) we added "**Remark 6** To illustrate the scalability of the proposed DDG method, an analysis is conducted from two aspects: computational burden and real-time feasibility.

1) Computational burden: Solving the local optimal control problem (Eq. 18-20) for each UAV involves a two-point boundary value problem with state dimension 8. We employ an efficient iterative solver (i.e., a gradient-based method) whose convergence per agent typically requires $\mathcal{O}(10^2)$ iterations in our simulations, with each iteration involving low-dimensional matrix operations [16]. The distributed architecture allows these computations to be parallelized across UAVs.

2) Real-time feasibility: For the inspection scenarios considered (mission duration 400s), the trajectory planning is computed offline or re-planned at low frequency (every 30s) based on updated neighbor states. The per-agent computation time (0.5s on a standard desktop CPU) is negligible compared to the re-planning interval, demonstrating the method's potential for near real-time operation.

We also compare our proposed approach with a centralized game-theoretic solver, which employs the same PMP principle and cost structure as our DDG method but solves a single, high-dimensional optimization problem using global information (i.e., centralized DG). This centralized solver must handle the concatenated state vector of all N UAVs, resulting in a total state dimension of $8N$. Consequently, its computational complexity scales approximately as $\mathcal{O}((N \times n)^3)$, where $n = 8$ represents the state dimension of a single UAV."

References

[16] Y. Li and X. Hu, A differential game approach to intrinsic formation control, Automatica 136 (2022) p. 110077.

RC: ***#2-5 - Lack of Reproducibility: Simulation settings, UAV parameters, cost matrices (Q, R, R_{ij}), and obstacle configurations are not fully specified.***

AR: Thank you for your raising this concern. We sincerely apologize for this oversight. To ensure full reproducibility, we have added Table 3(The other related simulation parameters of the proposed DDG.) in the Simulations section (Page 18), listing all critical parameters:

Table 3: The other related simulation parameters of the proposed DDG.

Parameter	Value
m_i	$1.5kg$
I_{ixx}	$0.03kg \cdot m^2$
I_{iyy}	$0.03kg \cdot m^2$
I_{izz}	$0.06kg \cdot m^2$
L_i	$0.15m$
$\hat{b}_i$	$1.5 \times 10^{-5} N \cdot s^2$
$\hat{d}_i$	$2.5 \times 10^{-7} N \cdot m \cdot s^2$
g_i	$9.81 m/s^2$
$\hat{R}_i$	$4m$
v_{imax}	$10m/s$
u_{imin}	$[0, -1, -1, -1]^T N$
u_{imax}	$[30, 1, 1, 1]^T N$
$v_i(0)$	$[0, 2, 0, 0]^T m/s$
Q_i	$0.1 \times I_8$
R_i	I_4
R_{ij}	$0.05 I_4$
F_i	$10 I_8$
$\hat{\alpha}_1$	0.3
$\hat{\alpha}_2$	0.1
$\bar{R}_i$	$20m$
Stopping criterion	$\|\tilde{z}_i\| \leq 0.01$

The complete MATLAB code implementing the DDG framework, benchmark methods, and the exact simulation environment (including all obstacle positions) is available in https://github.com/xuewenyan6-debug/Wenyan-Xue.git.

RC: ***#2-6- Language is mostly clear, but the manuscript needs polishing for grammar (e.g., "This model do not..." → "This model does not...").***

AR: We sincerely thank you for highlighting language and grammatical issues. We have now thoroughly revised the entire manuscript to correct grammatical errors, improve sentence structure, and enhance clarity. For example:“ This model do not account for. . . ” has been corrected to ”This model does not account for $\cdots$”.

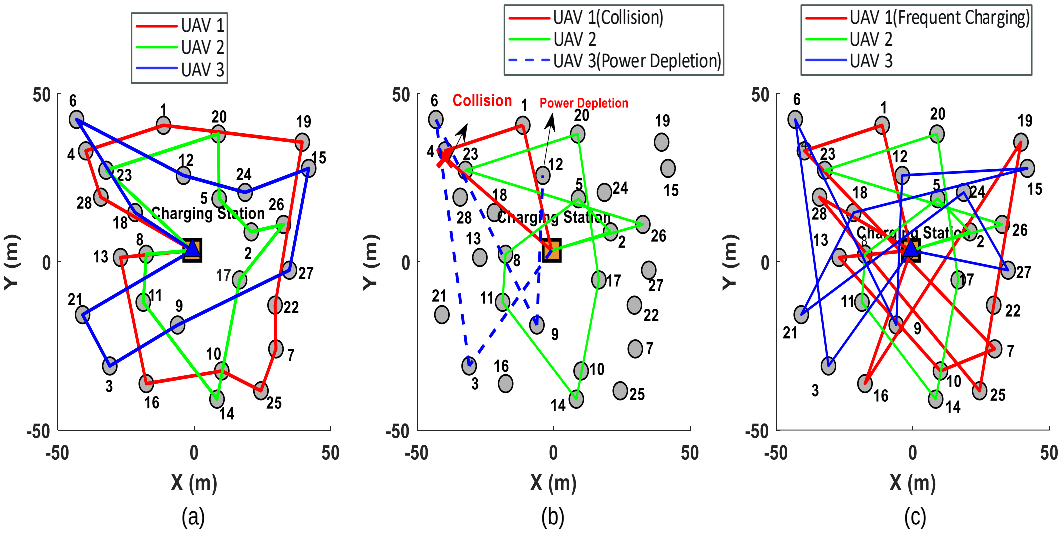

Figure 11: Comparison of inspection trajectories for 3 UAVs and 28 turbines: (a) Proposed DDG method: All UAVs (UAV1-3) complete inspection tasks with coordinated, collision-free paths and return to the OBS. (b) GA-DZ method [6]: UAV1 collides with Wind Turbine 4 (marked by 'X'), and UAV3 exhausts its battery before returning. (c) NN-DRL method [9]: All tasks are completed safely, but UAV1 exhibits an inefficient, elongated path requiring mid-mission returns to the OBS, increasing total time.

RC: ***#2-7- Figures lack proper resolution and labels (especially Fig. 3 and 4).***

AR: We sincerely appreciate your observation regarding the figure quality. In response, we have regenerated all figures—including Fig. 3 and Fig. 4—with higher resolution and enhanced clarity. Specifically, in Figure 11(Fig. 3 in the revised paper), we have added explicit axis labels (e.g., "X coordinate (m)" and "Y coordinate (m)"), incorporated a distinct legend to differentiate between UAVs and methods, and improved color contrast and line styles for better visual interpretation. For Figure.12(Fig. 4 in the revised paper), we have clearly labeled the axes as "Time (s)" and "Minimum distance (m)," included horizontal reference lines to indicate safe distance thresholds, and refined the legend and caption to improve readability and informational clarity. All figures are now prepared in high-resolution formats suitable for publication.

RC: ***#2-8 - Several variables are introduced before being defined. Maintain clear notation flow.***

AR: Thank you for pointing out this inconsistency. We have reorganized the notation and variable definitions throughout the manuscript to ensure a logical flow. Specifically: 1) All variables are now defined before their first use in mathematical expressions. 2) A consolidated Notation Table has been added in the Preliminaries section for quick reference (in the revised paper, page 4, Table 1). This restructuring improves readability and helps readers follow the mathematical development more easily.

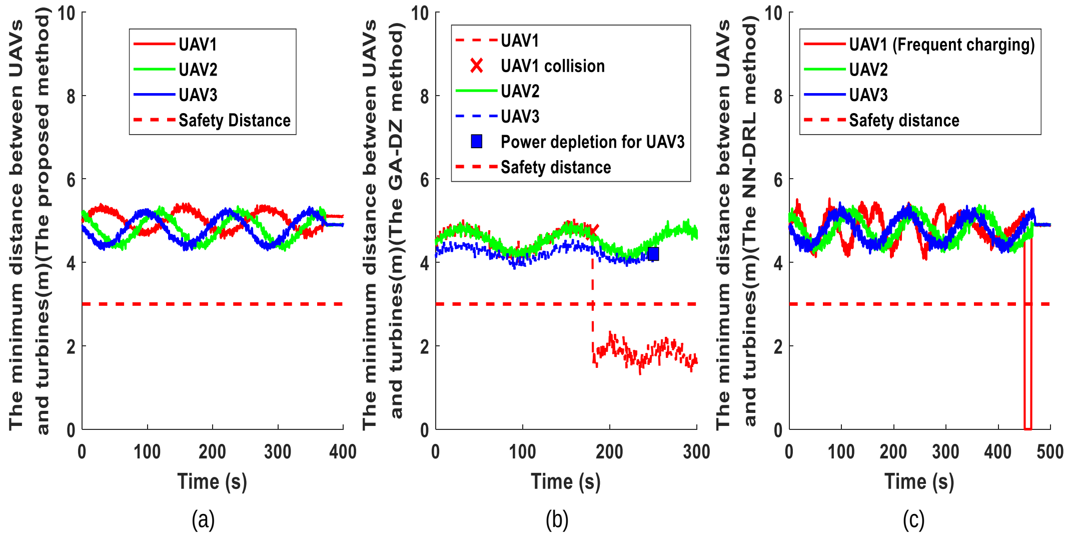

Figure 12: 3 UAVs and 28 turbines:(a)The minimum distance between UAVs and wind turbines with the proposed method (b)The minimum distance between UAVs and wind turbines with the GA-DZ method (c)The minimum distance between UAVs and wind turbines with the NN-DRL method.

RC: ***#2-9 - References are current but rely heavily on the authors' previous work; diversify citations.***

AR: We agree with your suggestion and have now expanded and diversified the reference list. While our previous work provided a foundational basis for this study, we have incorporated a broader range of recent and relevant literature from other research groups to better contextualize our contributions. Specifically, we have added recent works on UAV trajectory planning utilizing consensus algorithms [11], game-theoretic approaches [16-18,23], and model predictive control [15] developed by other authors. Additionally, key publications on offshore wind farm inspection, multi-agent systems, and distributed control have been included (see revised manuscript, Page 2). "Distributed optimization methods, such as based on consensus algorithm [11], have been proposed to alleviate reliance on a central coordinator, yet it often require iterative communication and may not explicitly account for dynamic collision avoidance or energy constraints in time-critical missions. ";"Recent efforts have explored hybrid centralized-distributed architectures [13] to balance optimality and robustness, but the fundamental issue of guaranteeing global performance with strictly local interactions persists.";"Alternatively, distributed model predictive control (DMPC) has been applied to handle unexpected changes in dynamic environments [15]. It synthesizes a state-feedback control strategy using a receding-horizon scheme. While DMPC can iteratively seek locally optimal solutions at each sampling instant, its focus on algorithmic optimization often prioritizes certain global objectives at the expense of individual UAV performance, which may lead to extended overall mission durations.

To bridge this gap, differential game theory has emerged as a promising framework for modeling multi-agent strategic interactions under dynamics constraints. It has been applied to various multi-robot coordination problems, such as formation control [16] and airborne conflict resolution [17]. This framework reconciles individual and collective objectives from a game equilibrium perspective,

providing a theoretical foundation for analyzing system-level outcomes. Specific to distributed settings, recent work has investigated graphical differential games for networked systems with limited communication [18, 19], establishing convergence to local Nash equilibria (L-NE) under certain connectivity conditions. Building upon this, Refs. [20, 21] advanced multi-player differential game solutions via a framework merging distributed optimal control with game theory, specifically addressing collision avoidance.

However, despite these advances, the direct application of existing differential game formulations to optimal trajectory planning for offshore wind turbine inspection poses distinct challenges, particularly under the stringent constraints of limited sensing, communication, and most critically, finite energy for round-trip missions. The main obstacles are as follows: 1) The NE derived in many generic multi-agent games or even in existing offshore wind inspection scenarios do not effectively enhance operational efficiency for battery-constrained UAVs, as round-trip energy constraints are seldom incorporated into the cost function design [22, 23]. "

The revised reference list now better reflects the current state of the field and acknowledges a wider range of scholarly contributions.

References

[11] Heinze J, Schopferer S, de Haag MU. Trajectory planning for offshore wind farm logistics with unmanned aircraft. In: 2024 AIAA DATC/IEEE 43rd Digital Avionics Systems Conference (DASC). IEEE; 2024. p. 1-10.

[13] Xuan L, Yang P, Lin Z, Yan P, Dong S, Dong J, et al. Research on design and control method of lightning protection detection robot for wind turbine. In: 2023 5th International Conference on Robotics and Computer Vision (ICRCV). IEEE; 2023. p. 41-5.

[15]Kong S, Liu Y. Model predictive control for risk-based inspection and maintenance planning of offshore wind turbines. Ships and Offshore Structures. 2025:1-12.

[16] Y. Li and X. Hu, A differential game approach to intrinsic formation control, Automatica 136 (2022) p. 110077.

[17] Rattanakul S, Chansiri K. Analysis of Strategic and Tactical Conflict Management for Urban Air Mobility and UAS in High-Density Airspace. Journal of Theoretical and Experimental Scientific Advancement. 2025;15(10):1-15.

[18] Yu D, Ge SS, Li D, Wang P. Finite-horizon robust formation-containment control of multi-agent networks with unknown dynamics. Neurocomputing.2021;458:403-15.

[19] Cappello D, Garcin S, Mao Z, Sassano M, Paranjape A, Mylvaganam T. A hybrid controller for multi-agent collision avoidance via a differential game formulation. IEEE Transactions on Control Systems Technology. 2020;29(4):1750-7.

[20] Xue W, Zhan S, Wu Z, Chen Y, Huang J. Distributed multi-agent collision avoidance using robust differential game. ISA transactions. 2023;134:95-107.

[21] Xu Y, Yang H, Jiang B, Polycarpou MM. A Games-in-Games Framework for Task Allocation, Path Planning, and Formation Control. IEEE Transactions on Control of Network Systems. 2024;12(1):620-33.

[22] Xue W, Zhan S, Chen N, Huang J. Optimal coordination in logistics warehousing with sensing

and communication limits: A distributed differential game approach. Asian Journal of Control. 2024;26(1):419-35.

[23] Singh SK, Reddy PV, Vundurthy B. Study of multiple target defense differential games using receding horizon-based switching strategies. IEEE Transactions on Control Systems Technology. 2021;30(4):1403-19."

3. Reviewer #3

3.1. General comments

RC: ***The paper tackles the problem of planning trajectories for a team of drones inspecting offshore wind turbines. The main challenges are: limited communication, avoiding collisions, and finite battery life, with a specific emphasis on making sure the drones can make it back to base. The authors frame this as a differential game where each drone is a player making decisions based only on local information from its neighbors. The core idea is to find a Nash Equilibrium for this game, and they make a theoretical argument that the local equilibrium found by their distributed method actually converges to a global one for the whole system. They compare their method against two others from the literature (a genetic algorithm approach and a neural network/deep RL approach) in simulation and show that their method is better at completing the inspection quickly and, crucially, ensures all drones return safely.***

-The use of a distributed differential game is a sophisticated and appropriate framework for this kind of multi-agent, conflicting-objectives problem. It is a step up from more basic optimization or learning techniques.

-The aspiration to prove convergence from a local to a global Nash equilibrium is commendable and, if solidly demonstrated, would be a valuable theoretical contribution.

-The simulation results, as presented, do seem to show a clear advantage over the chosen benchmarks, particularly in guaranteeing safety and complete mission success.

AR: We sincerely thank the reviewer for their insightful and constructive comments, as well as their recognition of the paper's motivation and potential contributions. We have carefully addressed each of the major concerns raised, as detailed below.

3.2. Major concern #3

RC: ***#3-1 - The Proof of Proposition 1 (Convergence of L-NE to G-NE). This is the theoretical heart of the paper, and I am afraid it is currently not convincing. The proof feels more like a sketch. My main confusion is with the matrix $\hat{\Omega}$. It appears in equation (25) seemingly out of nowhere, and its connection to the original Hamiltonian and the system dynamics is not explained. The entire argument hinges on $\hat{\Omega}$ being positive definite, but we are given no reason to believe this is true. The subsequent steps, which link the adjoint system's uniqueness to the global equilibrium, are stated but not rigorously derived. For the paper to fulfill its theoretical promise, this proof needs to be substantially expanded and clarified.***

AR: We agree with the reviewer that the original proof was overly concise and lacked clarity regarding the definition and properties of the matrix $\hat{\Omega}$. We sincerely thank the reviewer for this crucial observation. In the revised manuscript, we have added a detailed derivation of $\hat{\Omega}$ in the Appendix (pages 29 to 30 in the revised paper) and have substantially expanded and restructured the proof of Proposition 1 to enhance its rigor and transparency (pages 13 to 16 in the revised paper).

The detailed derivation of $\hat{\Omega}$ is as follows:"For the sake of clarity in the proof of the Proposition 1, we introduce the following detailed derivation of the adjoint system (33).

Consider a global cost function that consists of a weighted sum of the individual cost functions of each UAV:

$$J = \sum_{i=1}^{N} J_i. \tag{3}$$

Define the global Hamiltonian function:

$$H \triangleq \sum_{i=1}^{N} \left(\|Y_i(z_i)\|_{Q_i}^2 + \|u_i(t)\|_{R_i}^2 + \sum_{j \in \mathcal{N}_i} \|u_{ij}\|_{R_{ij}}^2 \right) + \lambda^T \left(Az + \sum_{i=1}^{N} \left(B_i u_i + \hat{\hat{g}}_i\right) \right). \tag{4}$$

where $\lambda \in \mathbb{R}^{8N}$.

According to the PMP, the optimal control u^* satisfies:

$$\frac{\partial H}{\partial u_i} = 0. \tag{5}$$

Then,

$$u_i^* = -R_i^{-1} B_i^T \lambda. \tag{6}$$

The state equation and costate equation are respectively:

$$\dot{z} = \frac{\partial H}{\partial \lambda} = Az + \sum_{i=1}^{N} \left(B_i u_i + \hat{\hat{g}}_i\right). \tag{7}$$

$$\dot{\lambda} = -\frac{\partial H}{\partial z} = -\sum_{i=1}^{N} \frac{\partial}{\partial z} \left(\|Y_i(z_i)\|_{Q_i}^2\right) - A^T \lambda. \tag{8}$$

We perform a quadratic approximation around the Nash equilibrium trajectory z^*, let $\hat{\hat{z}} = z - z^*$, then $Y_i(z_i)$ can be approximated as:

$$Y_i(z_i) \approx Y_i(z_i^*) + \nabla Y_i(z_i^*)^T \hat{\hat{z}}_i + \frac{1}{2} \hat{\hat{z}}_i^T \nabla Y_i^2(z_i^*)^T \hat{\hat{z}}_i. \tag{9}$$

Therefore,

$$\|Y_i(z_i)\|_{Q_i}^2 = \Xi + \hat{\hat{z}}_i^T \nabla Y_i(z_i^*) Q_i \nabla Y_i(z_i^*)^T \hat{\hat{z}}_i + \dots. \tag{10}$$

where Ξ is an constant.

Substituting the optimal control u^* (35) into the state equation (36), we obtain:

$$\dot{z} = Az + \sum_{i=1}^{N} \left(-B_i R_i^{-1} B_i^T \lambda + \hat{\hat{g}}_i\right). \tag{11}$$

Let $B = [B_1, \cdots, B_N]$, $R = diag\{R_1, \cdot, R_N\}$. Then the above equation can be written as:

$$\dot{z} = Az + -BR^{-1}B^T\lambda + \hat{\bar{g}}. \tag{12}$$

where $\hat{\bar{g}} = \sum_{i=1}^{N} \hat{\bar{g}}_i$.

The costate equation (37) can rewritten as:

$$\dot{\lambda} = -\sum_{i=1}^{N} \frac{\partial}{\partial z}\left(\|Y_i(z_i)\|_{Q_i}^2\right) - A^T\lambda = -\hat{\Omega}\hat{\bar{z}} - A^T\lambda. \tag{13}$$

where $\hat{\Omega} = \sum_{i=N}^{N} \frac{\partial^2}{\partial z^2}\left(\|Y_i(z_i)\|_{Q_i}^2\right)|_{z=z^*}$ is the value of the Hessian matrix of the global cost function with respect to the state z, evaluated at the equilibrium point.

Assuming that $\hat{\bar{g}}$ does not vary significantly near the equilibrium point, the linearized coupled system is:

$$\begin{bmatrix} \dot{\hat{\bar{z}}}(t) \\ \dot{\lambda}(t) \end{bmatrix} = \begin{bmatrix} A & -BR^{-1}B^T \\ -\hat{\Omega} & -A^T \end{bmatrix} \begin{bmatrix} \hat{\bar{z}}(t) \\ \lambda(t) \end{bmatrix}, \tag{14}$$

Consider the multi-UAV system with dynamics simplified to single-integrator form under Assumption 1 (slow flight and small attitude angles)[28], then, $A = 0$, $B = I$, for the special case where $R = I_{4N}$ (uniform control penalties), this simplifies to:

$$\begin{aligned} \begin{bmatrix} \dot{z}(t) \\ \dot{\lambda}(t) \end{bmatrix} &= \begin{bmatrix} 0 & -I_{8N} \\ -\hat{\Omega} & 0 \end{bmatrix} \begin{bmatrix} z(t) \\ \lambda(t) \end{bmatrix}, \\ \begin{bmatrix} z(0) \\ \lambda(t_f) \end{bmatrix} &= \begin{bmatrix} z(0) \\ Fz(t_f) \end{bmatrix}, \end{aligned} \tag{15}$$

Therefore, the matrix $\hat{\Omega}$ is essentially the value of the Hessian matrix of the global cost function J with respect to the state, evaluated at the Nash equilibrium point. It reflects the curvature of the total system cost with respect to changes in the state near the equilibrium. Positive definiteness implies that the cost function is convex in the vicinity of the equilibrium, thereby ensuring that the equilibrium point is locally optimal."

The proof of Proposition 1 has been restructured as follows(pages 13 to 16 in the revised paper):"
Proof. The proof is divided into three steps: (1) the algorithm converges to an L-NE (satisfying the PMP); (2) strong connectivity enforces global consistency through gradient exchange; (3) convexity ensures that the local solution is unique, and thus globally unique.

Step 1 (Algorithm convergence and attainment of the L-NE): For each UAV i, integrate the dynamics (Eq. (4)) over $[0, t_f]$ using the current control strategy $u_i^{(k)}(t)$.

$$\begin{cases} \dot{z}_i^{(k)}(t) = a_i z_i^{(k)}(t) + b_{ii}u_i^{(k)}(t) + \hat{\bar{g}}_{ii}, \\ z_i^{(k)}(0) = z_{i0}. \end{cases} \tag{16}$$

To efficiently compute the gradient $\nabla_{u_i} J_i^{(k)}$, an adjoint variable $\lambda_i(t) \equiv \rho_i(t) \in \mathbb{R}^8$ is introduced, governed by the adjoint equation (which provides an efficient computation of the costate equation in (20)):

$$\begin{cases} -\dot{\lambda}_i^{(k)}(t) = a_i^T \lambda_i^{(k)}(t) + Q_i \frac{\partial}{\partial z}\left(\|Y_i(z_i)\|^2\right), \\ \lambda_i^{(k)}(t_f) = F_i\left(z_i^{(k)}(t_f) - z_{ib}\right). \end{cases} \tag{17}$$

After backward integration, the gradient is obtained from the partial derivative of the Hamiltonian:

$$\nabla_{u_i} J_i^{(k)}(t) = R_i u_i^{(k)}(t) + b_{ii}^T \lambda_i^{(k)}(t). \tag{18}$$

Each agent updates its strategy along the negative gradient direction:

$$u_i^{(k+1)}(t) = u_i^{(k)}(t) - \eta \cdot \nabla_{u_i} J_i^{(k)}(t). \tag{19}$$

where $\eta > 0$ is the step size. Updated strategies $u_i^{(k+1)}$ are then broadcast to all neighbors $j(j \in \mathcal{N}_i)$. Upon convergence, for all $i \in \mathbb{N}_{1:N}$,

$$\nabla_{u_i} J_i^{(\infty)}(t) = R_i u_i^*(t) + b_{ii}^T \lambda_i^*(t) = 0, \quad \forall t \in [0, t_f]. \tag{20}$$

According to PMP, this condition, together with the state equation, adjoint equation, and transversality condition, constitutes the first-order necessary condition for optimality. In the distributed setting, this implies that for each agent i, given the optimal strategies of its neighbors u_{-i}^*, the strategy u_i^* is a local minimizer of its individual cost (9), thereby satisfying the definition of a L-NE (8).

Step 2 (Global consistency enforced by strong connectivity): Assume the communication graph $G(\mathcal{V}, \varepsilon)$ is strongly connected. Suppose, for contradiction, that the L-NE strategies are not globally consistent.(i.e., there exist two disjoint subsets of UAVs whose locally optimal solutions are mutually incompatible given the global mission objectives.) Such inconsistency would manifest as a mismatch in the coupled cost terms via the R_{ij} terms in (9). For any adjacent UAVs i and $j(j \in \mathcal{N}_i)$, a strategy discrepancy would produce a non-zero gradient component:

$$\left\| \frac{\partial}{\partial u_i} \sum_{j \in \mathcal{N}_i} \|u_{ij}\|_{R_{ij}} \right\| \neq 0. \tag{21}$$

During the iterative process, this gradient information is exchanged among neighbors (Step3 of Algorithm 1). Strong connectivity guarantees that there exists a directed path from any agent i to any other agent l. Consequently, any local inconsistency (nonzero gradient) propagates through the entire network via successive neighbor-to-neighbor exchanges.

Define the global gradient norm as $\Phi^{(k)} = \sum_{i=1}^{N} \int_0^{t_f} \left\| \nabla_{u_i} J_i^{(k)}(t) \right\|^2 dt$. The gradient-descent update ensures that $\Phi^{(k)}$ is non-increasing with k. At convergence,

$$\lim_{k \to \infty} \Phi^{(k)} = 0. \tag{22}$$

Then,

$$\nabla_{u_i} J_i^{(\infty)}(t) = 0. \tag{23}$$

This global zero-gradient condition implies that not only each agent's own gradient vanishes, but also all coupled interaction terms (via R_{ij}) are balanced, thereby eliminating any pairwise strategic contradictions. Hence, the locally optimal strategies u_i^* are globally consistent.

Step 3(Uniqueness and attainment of the G-NE): To establish the uniqueness of the G-NE, we begin by analyzing the adjoint system derived from a quadratic approximation of the problem around the equilibrium trajectory. Consider the Nash equilibrium trajectory z^*, Defining the deviation as $\hat{\tilde{z}} = z - z^*$, we have the collective state vector $\hat{\tilde{z}} = [\hat{\tilde{z}}_1^T, \cdots, \hat{\tilde{z}}_N^T]^T \in \mathbb{R}^{8N}$ and the co-state vector $\lambda = [\lambda_1^T, \cdots, \lambda_N^T]^T \in \mathbb{R}^{8N}$. The associated adjoint system with two-point boundary values is given by (The derivation is detailed in Appendix A):

$$\begin{aligned} \begin{bmatrix} \dot{\hat{\tilde{z}}}(t) \\ \dot{\lambda}(t) \end{bmatrix} &= \begin{bmatrix} 0 & -I_{8N} \\ -\hat{\Omega} & 0 \end{bmatrix} \begin{bmatrix} \hat{\tilde{z}}(t) \\ \lambda(t) \end{bmatrix}, \\ \begin{bmatrix} \hat{\tilde{z}}(0) \\ \lambda(t_f) \end{bmatrix} &= \begin{bmatrix} \hat{\tilde{z}}(0) \\ Fz(t_f) \end{bmatrix}, \end{aligned} \tag{24}$$

where the matrix $\hat{\Omega} \in \mathbb{R}^{8N \times 8N}$ is a block-diagonal matrix, $\hat{\Omega} = diag\{\hat{\Omega}_1, \cdots, \hat{\Omega}_i, \cdots, \hat{\Omega}_N\}$. Each block is constructed from the Hessian of the running cost function for UAV i evaluated at the equilibrium:

$$\hat{\Omega} = \sum_{i=1}^{N} \alpha_i \nabla^2 Y_i(z_i^*). \tag{25}$$

where z_i^* is the state of UAV i at the Nash equilibrium, $\nabla^2 Y_i(z_i^*)$ is the Hessian matrix of Y_i at z_i^*, $\alpha_i > 0$ is a positive weighting coefficient. The aggregate matrix $\hat{\Omega}$ thus represents a weighted sum of the individual Hessians.

The positive definiteness of $\hat{\Omega}$ is crucial and follows from the construction of the running cost $Y_i(z_i)$. This cost combines a collision avoidance penalty $D(z_i)$ and a trajectory optimization term $DS(z_i(t))$. The penalty term $D(z_i)$ is designed to be convex and increasing outside a safe distance, with a positive definite Hessian at the collision-free equilibrium point z_i^*. The trajectory term $DS(z_i(t))$ is also convex (e.g., based on squared angular deviation), yielding a positive semi-definite Hessian. By selecting positive coefficients α_i, the weighted superposition of these terms ensures that each $\nabla^2 Y_i(z_i^*)$ is positive semi-definite, with at least one block being positive definite. Consequently, the block-diagonal matrix $\hat{\Omega}$ is positive definite.

The positive definiteness of $\hat{\Omega}$ together with the positive definiteness of the weighting matrices Q_i, R_i, R_{ij}, F_i in the cost function (9), guarantees that the integrated cost term $\|Y_i(z_i)\|_{Q_i}^2 + \|u_i(t)\|_{R_i}^2 + \sum_{j \in \mathcal{N}_i} \|u_{ij}\|_{R_{ij}}^2$ and the terminal cost $\psi(z_i(t))$ are jointly convex. Therefore, given the strategies of its neighbors u_{-i}^*, each UAV's optimization problem is strictly convex. For a strictly convex problem, any point satisfying the first-order necessary condition (i.e., the zero-gradient condition) is the unique global minimizer. Thus, the strategy u_i^* obtained in Step 1 is the unique optimal response of UAV i to u_{-i}^*, each UAV's optimization subproblem is strictly convex. For a strictly convex problem, any point satisfying the first-order necessary optimality conditions (i.e., the zero-gradient condition derived from Pontryagin's Minimum Principle) is the unique global minimizer. Hence, the

strategy u_i^* obtained upon convergence of the distributed gradient algorithm is the unique optimal response of UAV i to its neighbors' strategies $u_{\hat{i}}^*$.

As established in Step 2 of the main proof, the strong connectivity of the communication graph $G(\mathcal{V}, \varepsilon)$, ensures that the locally optimal strategies are globally consistent. The collection of these unique local optimal responses, $u^* = (u_1^* \cdots u_i^* \cdots u_N^*)$, therefore forms a strategy profile that satisfies the definition of a G-NE (Definition 1). Formally, for every UAV i, if all other UAVs adhere to $u_{\hat{i}}^*$ (the strategies of all agents except i), then u_i^* is its optimal response:

$$J_i(z(0), u_i^*, u_{\hat{i}}^*) \leq J_i(z(0), u_i, u_{\hat{i}}^*), \quad u_i^* \neq u_i. \tag{26}$$

The distinction between the L-NE and the G-NE is that the G-NE considers the strategies of all other UAVs, not just immediate neighbors. The strong connectivity of the network, which enables the propagation of local consistency, guarantees the equivalence between these two notions in our framework. Consequently, the uniqueness of the solution to the adjoint system (ensured by $\hat{\Omega} > 0$), combined with the strong connectivity of $G(\mathcal{V}, \varepsilon)$, secures the convergence of the algorithm to a G-NE:

$$J_i\left(z(0), u_i^*, u_{-i}^*\right) = J_i(z(0), u_i^*, u_{\hat{i}}^*), \quad \forall i \in \mathbb{N}1 : N. \tag{27}$$

In summary, under a strongly connected communication topology, the distributed gradient algorithm converges to a strategy profile that is both a local and a global Nash Equilibrium.

□

References

[28] Zhou S, Hua H, Dong X, Li Q, Ren Z. Air-ground time varying formation tracking control for heterogeneous UAV-UGV swarm system. Aero Weaponry. 2019;26(4):54-9.

RC: ***#3-2 - Numerical Solution of the Two-Point Boundary Value Problem. The paper is almost completely silent on how the rather complex optimal control problem is actually solved. Equations (20) and (21) describe a TPBVP that is notoriously difficult to solve numerically, especially for a nonlinear, multi-agent system. The manuscript gives no information on the numerical methods used. The absence of these details makes it impossible to reproduce the results and leaves a major gap in the methodology. It also raises the question of computational burden, which is an important practical consideration for real-time application.***

AR: We thank the reviewer for highlighting this important methodological omission. The numerical solution of the TPBVP is indeed critical for implementing the proposed framework. We have added a new subsection titled "Distributed Dradient Optimization for L-NE" in the revised manuscript to describe the solution process transparently.

Distributed Dradient Optimization for L-NE

Solving the coupled TPBVP (20) directly in a distributed manner is challenging. Inspired by the Ref.[16] for solving TPBVP, Instead, we adopt a direct optimization approach: each UAV i iteratively improves its control trajectory $u_i(t)$ to minimize J_i directly, using only local information. This approach is numerically robust and naturally parallelizable.

We employ a distributed gradient-based optimization algorithm, summarized in the following

Algorithm 1. The control trajectory is parameterized over $[0, t_f]$. Each iteration involves: 1) Forward simulation: Integrate the dynamics (Eq. (4)) with the current control to obtain the state trajectory. 2) Gradient computation: Compute the gradient $\nabla_{u_i} J_i$ efficiently using the adjoint method. This requires a backward integration of an adjoint equation, which is computationally inexpensive and avoids explicit solution of the costate equation in (20). 3) Gradient update and communication: Update u_i along the negative gradient direction, then exchange the updated control with neighbors.

Remark 1 *Upon convergence, the solution obtained by the gradient-based optimization algorithm satisfies the first-order necessary conditions for a minimum of the cost functional J_i. These conditions are mathematically equivalent to the set of Pontryagin's Minimum Principle (PMP) conditions given in Eq.(20). Specifically, the adjoint variable $\rho_i(t)$ introduced in the gradient computation obeys the same linear differential equation and terminal condition as the PMP costate $\lambda_i(t)$. Because both variables satisfy an identical linear boundary-value problem, the uniqueness theorem for such problems guarantees that $\rho_i(t) \equiv \lambda_i(t)$ at convergence. Consequently, the trajectories generated by our distributed gradient algorithm fulfill all PMP necessary conditions and therefore constitute a L-NE.*

Even when the underlying system dynamics are nonlinear, the costate (or adjoint) equation remains a linear differential equation in $\lambda_i(t)$ (or $\rho_i(t)$). This linearity follows from the fact that the equation is derived either by linearizing the original Hamiltonian system around the optimal trajectory or directly from the variational principle. Hence, the uniqueness argument holds in the general nonlinear setting, ensuring the equivalence between the numerical solution of the gradient algorithm and the analytical PMP formulation.

The pseudo-code of the distributed gradient optimization for L-NE is as follows.

Algorithm 1 Distributed gradient optimization for L-NE

Input: Initial state $z_i(0)$, neighbor strategies u^*_{-i}, cost weights, horizon t_f, step size η_0, tolerance ϵ.
Output: Optimal control u_i^*..

Repeat
Forward simulation Integrate the dynamics $\dot{z}_i = a_i z_i + b_{ii} u_i^{(k)} + \hat{\hat{g}}_{ii}$ (Eq.4) forward from $t = 0$ to t_f, obtaining the state trajectory $z_i^{(k)}(t)$.
Gradient computation Compute the cost gradients $\frac{\partial L_i}{\partial z_i}$ and $\frac{\partial L_i}{\partial u_i}$ along $z_i^{(k)}(t)$.
Integrate the adjoint equation backward in time: $-\dot{\rho}_i = a_i^T \rho_i + \frac{\partial L_i}{\partial z_i}$ with terminal condition $\rho_i(t_f) = \frac{\partial \psi}{\partial z_i(t_f)}$.
Compute the gradient: $\nabla_{\mathbf{u}_i} J_i^{(k)} = \int_0^{t_f} \left(b_{ii}^T \rho_i(t) + \frac{\partial L_i}{\partial u_i} \right) dt$.
Distributed communication Broadcast the updated control $\mathbf{u}_i^{(k)}$ to all neighbors $j \in \mathcal{N}_i$.
Receive neighbors' controls $\mathbf{u}_j^{(k)}$ for $j \in \mathcal{N}_i$.
Projected gradient update
$\mathbf{u}_i^{(k+1)} \leftarrow \mathcal{P}_{\mathcal{U}} \left[\mathbf{u}_i^{(k)} - \eta_k \cdot \nabla_{\mathbf{u}_i} J_i^{(k)} \right]$, where $\mathcal{P}_{\mathcal{U}}$ projects onto the feasible control set.
Update step size η_k via backtracking line search.
$k \leftarrow k + 1$.
Until $\|\nabla_{\mathbf{u}_i} J_i^{(k)}\| < \epsilon$ for all i **or** $k > K_{\max}$.

The L-NE strategy u_i^* is obtained by numerically minimizing the cost function J_i in (9) subject to

the dynamics constraint (4). This is achieved using a distributed gradient descent algorithm, which directly optimizes the control trajectory without explicitly solving the two-point boundary value problem for the costate $\lambda_i(t)$.

The Pontryagin's Minimum Principle (PMP) applied to our DDG formulation yields the set of necessary conditions for optimality (Eqs. 20-21). These conditions, which include a two-point boundary value problem, define what constitutes a Local Nash Equilibrium (L-NE). The theoretical contribution of our work (Proposition 1) is to prove that under a strongly connected graph, the unique solution satisfying these local conditions for all agents converges to a Global Nash Equilibrium (G-NE).

Remark 2 *To illustrate the scalability of the proposed DDG method, an analysis is conducted from two aspects: computational burden and real-time feasibility.*

1) Computational burden: Solving the local optimal control problem (Eq. 18-20) for each UAV involves a two-point boundary value problem with state dimension 8*. We employ an efficient iterative solver (i.e., a gradient-based method) whose convergence per agent typically requires* $\mathcal{O}(10^2)$ *iterations in our simulations, with each iteration involving low-dimensional matrix operations [16]. The distributed architecture allows these computations to be parallelized across UAVs.*

2) Real-time feasibility: For the inspection scenarios considered (mission duration 400s), the trajectory planning is computed offline or re-planned at low frequency (every 30s) based on updated neighbor states. The per-agent computation time (0.5s on a standard desktop CPU) is negligible compared to the re-planning interval, demonstrating the method's potential for near real-time operation.

We also compare our proposed approach with a centralized game-theoretic solver, which employs the same PMP principle and cost structure as our DDG method but solves a single, high-dimensional optimization problem using global information (i.e., centralized DG). This centralized solver must handle the concatenated state vector of all N *UAVs, resulting in a total state dimension of* $8N$*. Consequently, its computational complexity scales approximately as* $\mathcal{O}((N \times n)^3)$*, where* $n = 8$ *represents the state dimension of a single UAV.*

References

[16] Y. Li and X. Hu, A differential game approach to intrinsic formation control, Automatica 136 (2022) p. 110077.

RC: ***#3-3 - Clarity of Presentation and Figures. The presentation significantly hampers understanding.***

-The schematic in Figure 1 is too small. The caption does not explain what the blocks represent or how information flows between them. A more detailed diagram and a self-explanatory caption are essential.

-In Figure 3, it's hard to see why one inspection trajectory is better than another.

-In Figure 4, It is not clear how to evaluate the curves and how to compare them among the different methods. Only in (b) it seems that the green line have failed.

-The description of the simulation setup is sparse. Communication range, how the task allocation was done initially, and the precise parameters for the benchmarks are all missing details that would

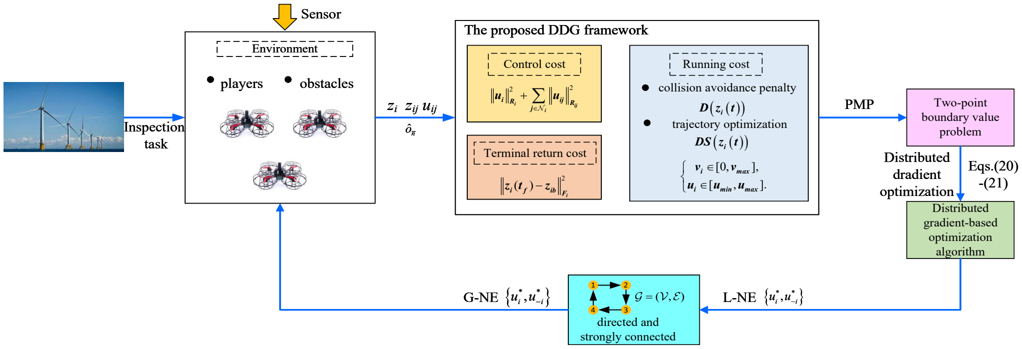

Figure 13: The schematic diagram of the proposed DDG framework.

help in judging the fairness of the comparison.

AR: We appreciate these suggestions for improving clarity and reproducibility. We have comprehensively revised all figures and expanded the simulation description.

The Figure 1 has been completely redrawn at a higher resolution with a clearer layout. To enhance our presentation, in the paper (page 7), we added "Each UAV i operates as an autonomous player. It receives local sensor data (own state z_i) and communicated information from neighbors $j \in \mathcal{N}_i$ (states z_{ij}, strategies u_{ij}). These inputs feed into its local Game Solver (green block), which solves the TPBVP (Eqs. (20)-(21)) via the numerical method (i.e., distributed dradient optimization for L-NE) to compute its optimal strategy u_i^*. This strategy is applied to its dynamics and also broadcast to its neighbors, closing the distributed feedback loop. The cyan block indicates information flow limited by the communication graph $G(\mathcal{V}, \varepsilon)$."

For Figure 3 (Trajectories), we have separated the subfigures (a), (b), and (c) into three distinct, larger plots to improve clarity. Each plot now includes: (i) a clear legend distinguishing the UAVs (UAV1–UAV3); (ii) labels for key points such as the Base and individual wind turbines; and (iii) visual collision markers where relevant. Furthermore, a supplementary table (in the revised paper, page 20, Table 4) has been added to statistically compare the inspection sequences and completion times of each UAV under the three methods. The figure caption has also been expanded to provide a detailed description of the trajectories(in the revised paper, page 18).

These enhancements make it immediately visually apparent that our proposed method (a) produces conflict-free and efficient inspection paths. In contrast, the GA-DZ method (b) results in a collision and an incomplete path, while the NN-DRL method (c) exhibits inefficient looping, thereby increasing the total inspection time.

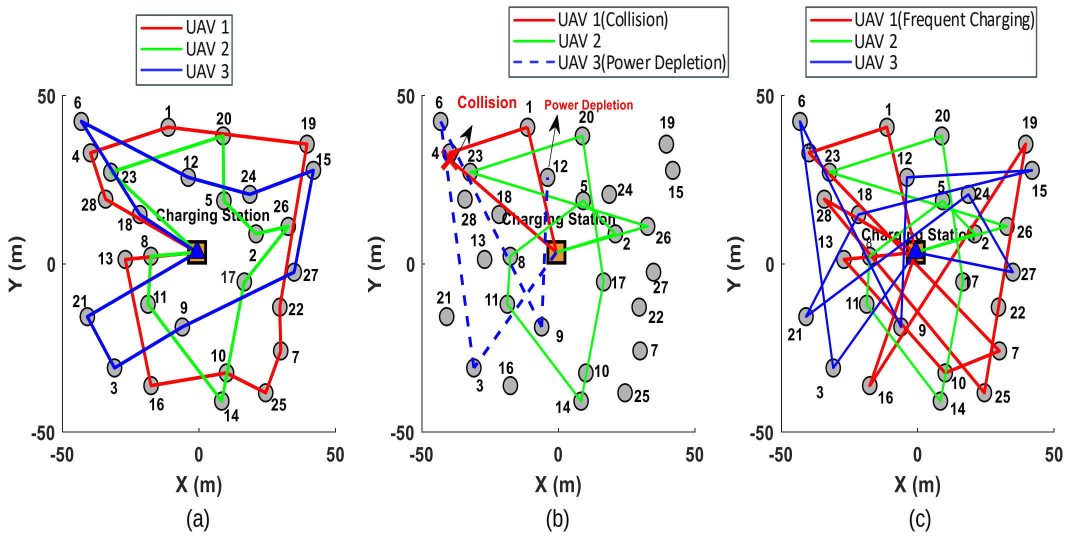

Figure 14: Comparison of inspection trajectories for 3 UAVs and 28 turbines: (a) Proposed DDG method: All UAVs (UAV1-3) complete inspection tasks with coordinated, collision-free paths and return to the OBS. (b) GA-DZ method [6]: UAV1 collides with Wind Turbine 4 (marked by 'X'), and UAV3 exhausts its battery before returning. (c) NN-DRL method [9]: All tasks are completed safely, but UAV1 exhibits an inefficient, elongated path requiring mid-mission returns to the OBS, increasing total time.

Table 4: The inspection sequences and path length with the three methods.

Method	Inspection sequences
The proposed method	UAV1:$\{28 \to 4 \to 1 \to 19 \to 22 \to 7 \to 25 \to 10 \to 16 \to 13\}(311m)$
	UAV2: $\{23 \to 20 \to 5 \to 2 \to 26 \to 17 \to 14 \to 11 \to 8\}(278m)$
	UAV3: $\{18 \to 6 \to 12 \to 24 \to 15 \to 27 \to 9 \to 3 \to 21\}$ $(256m)$
The GA-DZ method	UAV1: $\{1 \to 4\}$(Collision)$(102mm)$
	UAV2: $\{2 \to 26 \to 23 \to 20 \to 17 \to 14 \to 11 \to 8 \to 5\}(325m)$
	UAV3: $\{3 \to 6 \to 9 \to 12\}$ (Power depletion)$(136m)$
The NN-DRL method	UAV1: $\{13 \to 10 \to 7 \to 4 \to 1 \to Charging \to 16 \to 19 \to 22 \to 25 \to Charging \to 28\}(457$[illegible]
	UAV2: $\{2 \to 26 \to 5 \to 8 \to 17 \to 14 \to 17 \to 20 \to 23\}(316m)$
	UAV3: $\{27 \to 24 \to 21 \to 18 \to 15 \to 12 \to 9 \to 6 \to 3\}(294m)$

As shown in Figure 3, which shows the inspection planned trajectories under three methods. UAV1 is responsible for inspecting wind turbine set $\{1, 4, 7, 10, 13, 16, 19, 22, 25, 28\}$; UAV2 for set $\{2, 5, 8, 11, 14, 17, 20, 23, 26\}$; and UAV3 for set $\{3, 6, 9, 12, 15, 18, 21, 24, 27\}$. For clarity, the inspection sequences with the three methods are summarized in a Table 4. The results indicate that the proposed method achieves the shortest total path length of 845 m. This is because the proposed method transforms the estimated planning of the UAVs into an optimally coordinated DDG model, obtaining a G-NE trajectory that balances maximum flight range and reduced inspection time. The GA-DZ method [6], which uses a genetic algorithm to optimize trajectories and aims to reduce inspection time, neglects the safety constraints of the UAVs. This makes it unsuitable for densely distributed wind turbine scenarios. During inspection, the wind turbines remain operational. When UAV1 using the GA-DZ method flies from turbine 1 to turbine 4, it collides with turbine 4 due to unaccounted dynamic obstacles, preventing completion of subsequent tasks (Figure 3(b), red solid line). Additionally, UAV3 fails to complete its inspection because it runs out of battery while flying from turbine 9 to turbine 12, as the return energy constraint is not considered (Figure 3(b), blue dashed line). The NN-DRL method [9] yields the longest total path length of 10679 m. This is attributed to its tendency to fall into local optima during online trajectory planning. Although it considers the return energy constraint, frequent returns for recharging reduce inspection efficiency. For instance, UAV1 returns to charge three times, significantly increasing the total path length (Figure 3(c), red solid line).

For Figure 4 (Minimum Distances), we have implemented several key enhancements to improve clarity and interpretation: a horizontal dashed red line has been added to all subplots to clearly mark the safe distance threshold (4m); the Y-axis label is now specified as "Minimum Distance Between UAVs and Wind Turbines (m)"; in subplot (b) for the GA-DZ method, the violation where UAV1's trajectory (black curve) dips below the safety line is explicitly marked with an "X" and annotated as "collision" in the legend; furthermore, in subplot (c) for the NN-DRL method, a solid red line at the zero-distance point is introduced to clearly indicate the moments when UAV1 returns to base for recharging. These modifications collectively make the safety performance and operational inefficiencies of each method immediately apparent and quantifiable.

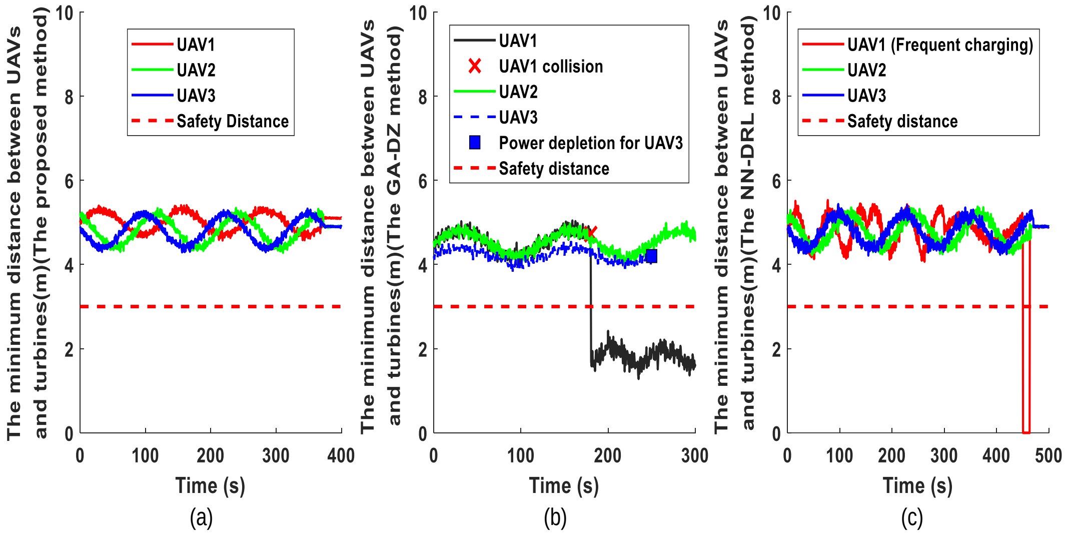

Figure 15: 3 UAVs and 28 turbines:(a)The minimum distance between UAVs and wind turbines with the proposed method (b)The minimum distance between UAVs and wind turbines with the GA-DZ method (c)The minimum distance between UAVs and wind turbines with the NN-DRL method.

To evaluate safety during inspection, Figure 4 shows the minimum distances between each UAV and obstacles (wind turbines and other UAVs within the field of view) for the three methods. Figure 4(a) corresponds to the proposed method, where all UAVs maintain distances greater than the safe threshold. Figure 4(b) illustrates the results for the GA-DZ method [6]. While UAV2 maintains safe distances, the minimum distance between UAV1 and wind turbine 4 falls below the safe threshold, indicating a collision. Moreover, UAV3 stops operating at 250 s due to battery depletion. Figure 4(c) presents the results for the NN-DRL method [9], where all UAVs maintain distances above the minimum safe level.

In this work, we assume that the communication relationship among UAVs follows a strongly connected and fixed communication topology, meaning that information exchange between a UAV and its neighbors is not constrained by physical distance. This assumption aligns with common practices in prior multi-agent coordination and control studies [1,2,3]. To better reflect real-world offshore wind farm inspection environments, future work will focus on the problem of multi-UAV game-theoretic inspection under time-varying communication topologies.

Furthermore, this paper focuses on how the assigned UAVs can safely complete their inspection missions, given that target wind turbines have already been allocated to each player. Thus, task allocation is not the central focus of this research. To ensure a fair comparison, all methods—both the proposed approach and the benchmark methods—are evaluated using the same initial task assignment for each UAV. All relevant parameters for these methods are provided in the paper (e.g., in the revised paper, pages 16 to 17, in Table 2 and Table 3), ensuring transparency and reproducibility.

To enhance the presentation, in the paper (page 16, Remark 8) we modified "**Remark 8** This study

focuses specifically on the operational control of multi-UAV systems during maritime inspection, and thus the simulations are confined to the operational space in which UAVs execute inspection tasks over offshore wind turbines. Given that target wind turbines are pre-assigned to each UAV, the core objective is to ensure the safe and efficient completion of these missions, rather than addressing the task allocation problem. To guarantee a fair comparison, all evaluated methods—including the proposed approach and the benchmarks—utilize the same initial task assignments and are implemented with fully disclosed parameters.

Furthermore, we added(in the revised paper, pages 16 to 18)"For the GA-DZ baseline, we adopted the genetic algorithm with dynamic zoning as outlined in Ref.[6], utilizing the authors' publicly available source code. The algorithm encodes solutions as sequences of waypoint assignments and corresponding flight trajectories. Its optimization is driven by a fitness function defined as the inverse of the total path distance for the UAV fleet. To ensure a meaningful comparison that tests the algorithm's inherent ability to satisfy constraints, substantial penalties (1×10^6 per violation) are applied in the fitness evaluation for any path that violates safety distances limits. The evolutionary process uses a population size of 100 and runs for 500 generations per simulation, with a crossover rate of 0.85, a mutation rate of 0.1, and tournament selection (size=3). Unlike learning-based methods, GA-DZ does not involve a separate training phase; it is executed directly on each test scenario to produce a scenario-specific solution, enabling a direct and fair performance comparison under the same conditions as the proposed DDG method.

For the NN-DRL baseline, we implemented a Dueling Deep Q-Network (DQN) following the architecture in Ref.[9]. The network takes the UAV's state as input, processes it through two fully-connected layers, and outputs Q-values for each discrete action. The state space includes the UAV's pose p_i, velocity v_i, remaining battery, relative positions to its target and the nearest wind turbine, as well as positions of neighboring UAVs within communication range. The action space is defined as nine discrete actions: hovering and moving in eight fixed-speed directions. The reward function is designed to balance multiple objectives and is formulated as:

$$\hat{r}_i = -\varkappa_1 d_i - \varkappa_2 \bar{e}_i + \varkappa_3 \hat{e}_i - \varkappa_4 \hat{c}_i - \varkappa_5 \hat{f}_i, \quad \forall i \in \mathbb{N}_{1:N}. \tag{28}$$

where d_i, $\bar{e}_i$, $\hat{e}_i$, $\hat{c}_i$ and $\hat{f}_i$ denote distance to target, energy consumption, inspection completion reward, collision penalty, and return violation penalty, respectively, with all coefficients tuned accordingly. During training, an ϵ-greedy exploration strategy is adopted, with ϵ linearly decaying from 1.0 to 0.05 over the first $8,000$ episodes. Each agent was trained for $10,000$ episodes on a randomized set of training scenarios to ensure strategy generalization. The trained strategy was then evaluated on a separate, held-out test set that is identical to the scenarios used for evaluating the proposed DDG method, thereby ensuring a fair comparison. The parameters of the NN-DRL method are summarized in Table 2.

Table 5: The parameters of the NN-DRL method [9].

Parameter	Value
Learning rate	0.001
Discount factor	0.99
Replay buffer size	50000
Batch size	64
Target update frequency	100$step$
Total number of training episodes	10000

We consider a multi-UAV inspection system comprising three UAVs (i.e., $N = 3$). The environment contains 28 offshore wind turbines and one offshore booster station (OBS), which serves as both the charging base and the common starting point for all UAVs. The simulated obstacles include known wind turbine towers, each with a radius of $5m$, as well as other UAVs, which are treated as unknown dynamic obstacles. Each UAV is assigned a specific subset of wind turbines for inspection and plans its trajectory accordingly. To ensure a fair comparison, the proposed DDG method and the two benchmark approaches [6, 9] are evaluated under identical conditions: the same initial positions and dynamics model (Eq. 3), the same environmental layout (28 turbines and 1 OBS), and the same success criteria (complete inspection, collision-free operation, and safe return to the OBS). This setup isolates the performance differences to the algorithmic level. All other relevant simulation parameters of the proposed DDG are summarized in Table 3.

Table 6: The other related simulation parameters of the proposed DDG.

Parameter	Value
m_i	$1.5kg$
I_{ixx}	$0.03kg \cdot m^2$
I_{iyy}	$0.03kg \cdot m^2$
I_{izz}	$0.06kg \cdot m^2$
L_i	$0.15m$
$\hat{b}_i$	$1.5 \times 10^{-5} N \cdot s^2$
$\hat{d}_i$	$2.5 \times 10^{-7} N \cdot m \cdot s^2$
g_i	$9.81m/s^2$
$\hat{R}_i$	$4m$
v_{imax}	$10m/s$
u_{imin}	$[0, -1, -1, -1]^T N$
u_{imax}	$[30, 1, 1, 1]^T N$
$v_i(0)$	$[0, 2, 0, 0]^T m/s$
Q_i	$0.1 \times I_8$
R_i	I_4
R_{ij}	$0.05I_4$
F_i	$10I_8$
$\hat{\alpha}_1$	0.3
$\hat{\alpha}_2$	0.1
$\bar{R}_i$	$20m$
Stopping criterion	$\|\tilde{z}_i\| \leq 0.01$

As shown in Figure 3, which shows the inspection planned trajectories under three methods. UAV1 is responsible for inspecting wind turbine set $\{1, 4, 7, 10, 13, 16, 19, 22, 25, 28\}$; UAV2 for set $\{2, 5, 8, 11, 14, 17, 20, 23, 26\}$; and UAV3 for set $\{3, 6, 9, 12, 15, 18, 21, 24, 27\}$. "

Reference

[1] Xu Y, Yang H, Jiang B, Polycarpou MM. A Games-in-Games Framework for Task Allocation, Path Planning, and Formation Control. IEEE Transactions on Control of Network Systems. 2024;12(1):620-33.

[2] Singh, Sharad Kumar and Reddy, Puduru Viswanadha and Vundurthy, Bhaskar. Study of

multiple target defense differential games using receding horizon-based switching strategies. IEEE Transactions on Control Systems Technology. 2021;30(4):1403-19.

[3] Wang X, Xiao Z, Ren Z, Dong C, Tian XD. Formation cooperative trajectory tracking control for unmanned aerial vehicles via differential game and reinforcement learning. Transactions of the Institute of Measurement and Control. 2025;47(9):1762-70.

[6] Peng Z, Sun S, Tong L, Fan Q, Wang L, Liu D. Optimization of offshore wind farm inspection paths based on K-means-GA. Plos one. 2024;19(5):e0303533.

[9] Fan T, Fu L, Guo C, Zhang Y, Sun L. Multi UAV Inspection Optimization for Offshore Wind Farms Considering Battery Exchange Process. IEEE Transactions on Intelligent Vehicles. 2024.

RC: ***#3-4 - The abstract says "enhances inspection efficiency and reducing task completion time" - it should be "reduces".***

AR: We sincerely thank the reviewer for catching this grammatical inconsistency. This has been corrected in the revised abstract. The sentence now reads: "$\cdots$ the method significantly enhances inspection efficiency and reduces task completion time by up to 18.7%$\cdots$".

RC: ***#3-5 - Table 1 is a bit strange and feels unnecessary. Also, reporting the mean convergence time for one successful run is not very statistically meaningful. It would be stronger to see results over multiple randomized trials.***

AR: We appreciate the reviewer's insightful comment regarding statistical rigor. The primary purpose of the original Table 1 was to succinctly summarize the key comparative outcome—whether the mission was completed and the relative time efficiency. We agree that presenting only the mean from a single successful run for our method is insufficient. we have removed the original Table 1 and significantly strengthened the results section. We now report outcomes from 30 randomized trials for each method. In each trial, the initial positions of the UAVs and the assignment sequence of wind turbines were varied within a defined operational range. A new Table 6 (Summary of Monte Carlo Simulation Results over 30 Trials) has been added.(in the revised paper, pages 23 to 25):"To further illustrate the efficacy in minimizing inspection time without compromising safety of the proposed DDG method, we conducted a comparative validation of the three methods in 30 randomly generated simulation scenarios. In each trial, the initial positions of the UAVs and the assignment sequence of wind turbines were varied within a defined operational range. Each scenario involves 3 UAVs and 28 wind turbines, with the inspection tasks per UAV and algorithm parameters remaining unchanged (see Table 2 to 3). Figure 10 visually presents the data distribution of the three methods in terms of minimum distance and task completion time. The figure includes the median (line inside the box), interquartile range (box range), whiskers (normal data range), and outliers (individual points), providing complete statistical distribution information.

By observing Figure 10(a), the minimum distance distributions of the proposed DDG method (median: 5.25 m, interquartile range: 4.64–5.89 m) and the NN-DRL method (median: 5.13 m, interquartile range: 4.58–5.69 m) are highly overlapping, and both are significantly above the 4.0 m safety distance (indicated by the dashed line). Both methods maintain a 100% success rate across the 30 scenarios. The box height of the DDG method is slightly narrower than that of the NN-DRL method, indicating better inter-scenario consistency in maintaining safe distance. All data points are above 4.5m, with no outliers. The GA-DZ method succeeded in only 6 scenarios (20% success rate), and although its minimum safe distance distribution (median: 4.13 m) is above the threshold, it contains multiple data points close to the lower limit. More importantly, 80% of the scenarios

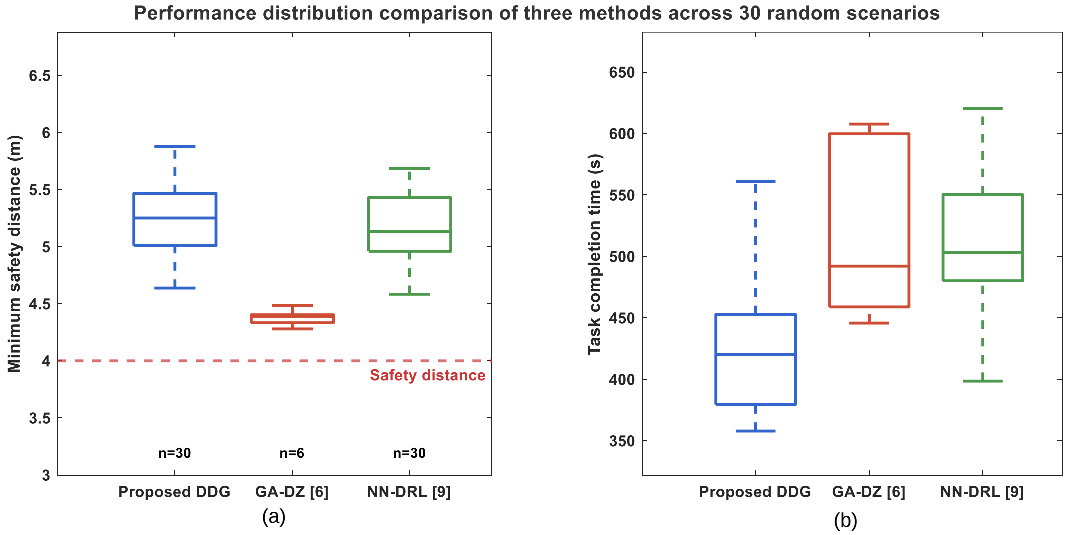

Figure 16: (a)The minimum distance between UAVs and wind turbines with the three methods (b)The task completion time between UAVs and wind turbines with the three methods.

failed due to collisions or energy depletion, confirming the high risk of this method in practical applications.

By observing Figure 10(b), the task completion time distribution of the proposed DDG method (median: 420.0 s, interquartile range: 357.9–561.0 s) is entirely lower than that of the NN-DRL method (median: 503.2 s, interquartile range: 398.6–620.4 s). The box of the DDG method is completely below that of the NN-DRL method, visually demonstrating its efficiency advantage. The average task completion time of the DDG method (381.2 ± 19.8 s) is 87.7 seconds shorter than that of the NN-DRL method (468.9 ± 23.5 s), representing a relative improvement of 18.7%. This improvement is reflected in the box plot as a clear vertical offset. The interquartile range of the DDG method (36.6 s) is narrower than that of the NN-DRL method (41.6 s), indicating lower sensitivity to different scenario configurations and better predictability in time. The GA-DZ method has valid data only in 6 successful scenarios (median: 492.4 s), but considering its 80% failure rate, its average task completion time is effectively infinite.

Table 7: The comprehensive statistical results using the three methods.

Performance metric	Proposed DDG method	GA-DZ Method [6]	NN-DRL Method [9]
Success rate	100% (30/30)	20% (6/30)	100% (30/30)
Minimum Safety Distance (m)	5.36 ± 0.29	4.13 ± 0.25	5.25 ± 0.42
Task Completion Time (s)	381.2 ± 19.8	∞ (80% failure)	468.9 ± 23.5

The relevant performance metrics from the 30 randomly generated simulation scenarios are sum-

marized in Table 7(Table 6 in the revised paper). Based on the comprehensive statistical analysis across 30 randomized scenarios, the proposed DDG method demonstrates significant and consistent advantages over the benchmark methods [6, 9]. While maintaining statistically equivalent safety performance (5.36 ± 0.29 m) and perfect reliability (100% success rate) compared to the NN-DRL method, it achieves a remarkable 18.7% reduction in task completion time (381.2 vs. 468.9 seconds). This combination of enhanced efficiency and unwavering reliability—standing in sharp contrast to the GA-DZ method, which fails in 80% of scenarios due to collisions or energy exhaustion—establishes the DDG framework as a highly efficient and robust solution for practical offshore wind farm inspection, particularly well-suited for resource-constrained UAV platforms."

RC: ***#3-6 - Are there other modern distributed control methods, such as distributed Model Predictive Control, that could be used for a more comprehensive comparison?***

AR: This is an excellent point regarding benchmarking. We thank the reviewer for raising it. In the original submission, we selected the GA-DZ [6] and NN-DRL [9] methods for two specific reasons: 1) They are contemporary and directly relevant benchmarks from recent literature focusing on offshore wind farm inspection using UAVs, allowing for a comparison within the same application domain. 2) They represent two dominant and distinct paradigms: offline heuristic optimization (GA) and online learning-based control (DRL). Comparing against these highlights the advantages of our game-theoretic, model-based approach which offers guaranteed equilibrium properties.

We fully agree that comparing against distributed model predictive control (DMPC)—a major paradigm in distributed control—would further strengthen the paper's contribution. Such a comparison would be highly valuable. Then, in the Introduction section (revised paper, page 2), we added"Alternatively, distributed model predictive control (DMPC) has been applied to handle unexpected changes in dynamic environments [15]. It synthesizes a state-feedback control strategy using a receding-horizon scheme. While DMPC can iteratively seek locally optimal solutions at each sampling instant, its focus on algorithmic optimization often prioritizes certain global objectives at the expense of individual UAV performance, which may lead to extended overall mission durations."

To ensure a fair comparison, we introduced collision avoidance and round-trip energy constraints consistent with those in our DDG framework into the DMPC model from Ref.[15], thereby adapting it to the inspection scenario designed in this study. The comparative results are presented in the simulation section (revised manuscript, pages 26–28):"

To further demonstrate the advantages of the proposed DDG method, a comparative analysis was conducted against the widely-used DMPC approach [15] under two operational paradigms: 3 UAVs inspecting 28 wind turbines and 6 UAVs inspecting 40 wind turbines. For each paradigm, 10 different random simulation scenarios were generated. The following performance metrics were statistically evaluated: the minimum safe distance between UAVs and obstacles (including turbines and other UAVs within sensing range), the total task completion time, and the on-board computation time. In the DMPC [15] implementation, the prediction horizon is set to 10 steps. To ensure a fair comparison between the algorithms, the cost function is kept consistent with that of the DDG approach, and the fmincon solver is employed for optimization. The statistical results are presented in Figure 17(Fig. 12 in the revised paper).

Figures. 17(a) and (d) illustrate that, in terms of safety, both methods successfully avoid collisions in all simulations, consistently maintaining obstacle-avoidance distances above the $4.0m$ safety threshold. This confirms that the DDG method preserves a safety level comparable to that of DMPC.

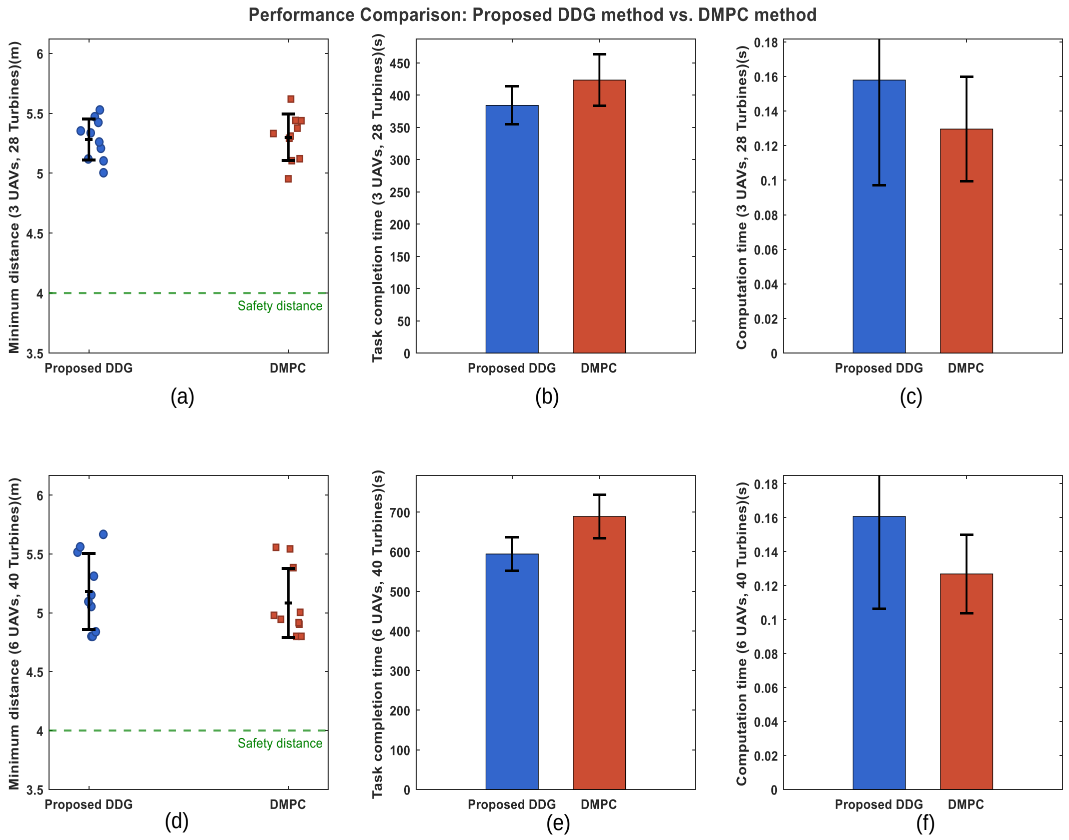

Figure 17: (a) The minimum distance for 3 UAVs inspecting 28 wind turbines with the two methods (b) The task completion for 3 UAVs inspecting 28 wind turbines with the two methods (c) The computation time for 3 UAVs inspecting 28 wind turbines with the two methods (d) The minimum distance for for 6 UAVs inspecting 40 wind turbines with the two methods (e) The task completion for 6 UAVs inspecting 40 wind turbines with the two methods (f) The computation time for 6 UAVs inspecting 40 wind turbines with the two methods.

In terms of task efficiency, as observed in Figures. 17(b) and (e), the DDG method reduces the average task completion time by approximately 12%. For instance, in the 6-UAV scenario, DDG achieves $580 \pm 70s$, whereas DMPC [15] requires $659 \pm 79s$. This improvement stems from the game-theoretic foundation of DDG, which explicitly models the strategic interactions among agents and drives the system toward a NE. Under the assumption of strong connectivity, the L-NE attained by DDG is guaranteed to be globally optimal, ensuring that each UAV's trajectory is globally balanced and coordinated. In contrast, DMPC [15] relies on algorithmic optimization to seek locally optimal solutions at each sampling instant. While such solutions may optimize certain global objectives, they often do so at the expense of individual agent performance, leading to longer overall mission times.

Regarding computational efficiency, Figures 12(c) and (f) demonstrate that DDG exceeds the on-board computation time of DMPC by 1.9% to 34.3%. In the 6-UAV scenario, DDG requires $0.165 \pm 0.650s$, whereas DMPC only demands $0.13 \pm 0.840s$. This significant difference stems from the fact that DDG needs to obtain the L-NE strategy through online game theory at each sampling instant. In contrast, DMPC solve an online optimization problem at every sampling interval, the prediction horizon interval of DMPC is shorter than the game-theoretic cycle of DDG at each sampling time, while DDG exhibits only a mild increase in computation, underscoring its superior scalability and real-time capability.

In summary, the proposed DDG method not only matches DMPC in safety assurance but also significantly outperforms it in task efficiency, even though its online computation time is slightly higher. Its game-theoretic formulation ensures globally balanced trajectories with low communication and computation demands, surpassing both the GA-DZ [6] and NN-DRL methods [9]. This makes it particularly suitable for distributed cooperative inspection in offshore wind farms, where communication is limited."

Reference

[6] Peng Z, Sun S, Tong L, Fan Q, Wang L, Liu D. Optimization of offshore wind farm inspection paths based on K-means-GA. Plos one. 2024;19(5):e0303533.

[9] Fan T, Fu L, Guo C, Zhang Y, Sun L. Multi UAV Inspection Optimization for Offshore Wind Farms Considering Battery Exchange Process. IEEE Transactions on Intelligent Vehicles. 2024.

[15] Kong, Siwen and Liu, Yan. Model predictive control for risk-based inspection and maintenance planning of offshore wind turbines. Ships and Offshore Structures. 2025:1-12.
